# Synergistic enhancement of soybean yield and quality by diethyl aminoethyl hexanoate: unraveling the molecular mechanisms through integrated transcriptomics

**DOI:** 10.3389/fpls.2026.1784831

**Published:** 2026-04-10

**Authors:** Qingnan Hao, Chao Li, Yuanxiao Yang, Shuilian Chen, Hongli Yang, Zhonglu Yang, Zhihui Shan, Haifeng Chen

**Affiliations:** 1 Oil Crops Research Institute, Chinese Academy of Agricultural Sciences, Wuhan, China; 2 Key Laboratory of Biology and Genetics Improvement of Oil Crops, Ministry of Agriculture and Rural Affairs, Wuhan, China

**Keywords:** carbon metabolism, diethyl aminoethyl hexanoate, leaf priming, plant hormone signaling, RNA-seq, soybean, yield-quality trade-off

## Abstract

Plant growth regulators like DA-6 can enhance crop productivity and stress tolerance, yet how DA-6 overcomes the common trade-off between yield and seed quality in soybean remains unclear. Using field experiments combined with multi-tissue transcriptomics and weighted gene co-expression network analysis (WGCNA) at key developmental stages, we demonstrate that DA-6 application increases soybean yield by 11.5–13.4% without compromising seed protein or oil content. Mechanistically, DA-6 spatiotemporally reprograms hormone signaling and carbon metabolism, inducing a “seed-like” program in leaves—including *de novo* expression of storage proteins and oleosins—effectively priming leaves as temporary nutrient reservoirs. During flowering, DA-6 elevates auxin responses and suppresses jasmonate signaling to favor vegetative growth; at pod-filling, it activates jasmonate, cytokinin, and MAPK pathways to enhance stress resilience. Stems are transformed into metabolic hubs through upregulated starch degradation, trehalose metabolism, and cell wall remodeling. WGCNA further linked MAPK signaling to amino sugar metabolism and endocytosis in yield-associated modules. These findings provide a molecular framework for DA-6–mediated synergistic improvement of yield and quality, revealing novel regulatory targets for crop optimization.

## 1 Introduction

Soybean (Glycine max [L.] Merr.) is a cornerstone of global agriculture, serving as a primary source of approximately 25% of the world’s edible oil and 70% of plant-derived protein for livestock feed (Liang et al., 2022). Despite its paramount agronomic importance, soybean productivity is frequently constrained by a combination of abiotic stresses—such as drought and salinity—and inherent inefficiencies in resource allocation during the critical seed-filling period (Sinclair and Purcell, 2005). While traditional breeding and agronomic interventions, including nitrogen fertilization, have been mainstays of yield improvement, their efficacy in simultaneously breaking the negative correlation between yield and seed quality is often limited. This underscores an urgent need for innovative strategies that can unlock greater yield potential without compromising nutritional value.

Plant growth regulators can precisely modulate crop development (Amoanimaa-Dede et al., 2022). DA-6 (diethyl aminoethyl hexanoate), a synthetic tertiary amine plant growth regulator with low molecular weight and high biological activity, has been shown to promote soybean growth and yield under field conditions. Wang et al. (2025) demonstrated that foliar application of 30–60 mg·L^−1^ DA-6 at flowering significantly increased soybean yield and enhanced soluble protein and sugar contents. Luo et al. (2021) reported that foliar application of 60 mg·L^−1^ DA-6 at flowering enhanced photosynthetic parameters, dry matter accumulation, and grain-filling duration in soybean, increasing yield by 36.7–38.4% in intercropping and 21.7–26.6% in monoculture systems.

Despite these documented physiological benefits, the molecular mechanisms by which DA-6 coordinates source-sink relationships to simultaneously enhance yield and quality under non-stress conditions remain uncharacterized. Functionally, DA-6 has been classified as a cytokinin-like substance that increases chlorophyll content and stimulates nucleic acid synthesis (He et al., 2014). Previous studies have also indicated that foliar application of DA-6 can enhance soybean yield and protein content (Jiang et al., 2012; Liu et al., 2019), hinting at its potential to coordinately regulate multiple traits.

The central challenge in modern soybean cultivation lies in achieving the simultaneous enhancement of yield and quality. A singular focus on yield improvement often leads to a dilution of seed protein and oil content, placing producers at a competitive disadvantage. Conversely, prioritizing quality traits frequently comes at the expense of yield, reducing overall cultivation profitability. Therefore, deciphering and overcoming this stubborn yield-quality trade-off is a critical objective for sustainable soybean production.

Intriguingly, foliar application of DA-6 appears to effectively break this negative correlation. However, the molecular mechanisms orchestrating this synergistic improvement remain unclear and demand in-depth investigation. To address this knowledge gap, we selected the widely cultivated soybean cultivar ‘Zhongdou 63’ and applied DA-6 at two critical developmental windows: R1 (beginning bloom) and R5 (beginning seed fill) stages, as defined by the standard soybean growth staging system (Fehr and Caviness, 1977). By integrating multi-tissue, multi-time-point transcriptomics with field physiological data and weighted gene co-expression network analysis (WGCNA), we systematically dissected the dynamic molecular network through which DA-6 coordinates the simultaneous enhancement of yield and quality. Our findings provide a robust molecular framework and identify potential regulatory targets for high-yield, high-quality soybean cultivation.

## 2 Materials and methods

### 2.1 Planting materials and field treatments

The soybean cultivar ‘Zhongdou 63’, extensively cultivated in Hubei Province, was used as the experimental material. Field experiments were conducted in June 2020 at two locations in Hubei Province, China: the Tuanslin Shuangbei Experimental Base of the Hubei Provincial Academician Workstation at Jingmen (China Agricultural Valley) Agricultural Science Research Institute, and the Yangluo Experimental Base of the Oil Crops Research Institute, Chinese Academy of Agricultural Sciences. The experimental fields featured clay soil with moderate fertility and flat terrain, where the preceding crop was rapeseed. Prior to sowing, compound fertilizer (N-P-K) was applied at a rate of 375 kg·ha-2. All other field management practices, including irrigation and pest control, followed conventional local farming protocols. A uniform experimental protocol was strictly implemented across all test sites.

The experiment employed a randomized complete block design with three independent biological replications. Seeds were drill-sown using string-guided precision planting with the following specifications: row length of 3 m, row spacing of 0.5 m, plant spacing of 0.1 m, five-row plots, and a total plot area of 7.5 m². Manual dibbling was performed with three seeds per hill, and seedlings were thinned to one plant per hill after emergence to ensure uniform plant density. Plants reached physiological maturity in late August and were manually harvested.

Two treatments were established: T0 (Control): Foliar application of water only at R1 and R5 stages, with a spray volume of 375 L·ha^−1^. T1 (DA-6 Treatment): Foliar application of 60 mg·L^−1^ DA-6 at both R1 and R5 stages, with the same spray volume of 375 L·ha^−1^.

Spraying was conducted using a handheld sprayer, and a non-ionic surfactant (Tween-20 at 0.1% v/v) was added to all solutions (both T0 and T1) to ensure even coverage and adhesion. Sampling for molecular analyses was conducted on sunny mornings between 8:00-11:00 AM at two weeks after DA-6 application.

### 2.2 Field data measurement methods and statistical analysis

At soybean maturity, 10 representative plants from the central area of each plot were sampled for indoor agronomic trait evaluation and subsequent quality analysis. The following parameters were meticulously measured:

Agronomic traits: Plant height, number of main stem nodes, number of productive branches per plant, total number of pods per plant, total seed weight per plant, and 100-seed weight. Mean values for each parameter were calculated and used as the plot-level trait measurement.

Quality traits: Seed protein and fat contents were determined using a Zeltex ZX50 portable near-infrared grain analyzer (Zeltex, USA), which was calibrated against standard wet chemistry methods prior to use.

Yield measurement: The central three rows of each plot were manually harvested, threshed, and the seeds were cleaned and weighed to determine the final grain yield, which was adjusted to a standard moisture content of 13%.

### 2.3 Statistical analysis

Data were subjected to analysis of variance (ANOVA) using SPSS Statistics software (Version 26, IBM Corp.). Treatment means were compared using the least significant difference (LSD) test at a significance level of P < 0.05.

### 2.4 Tissue collection and RNA extraction

To capture systemic transcriptional reprogramming induced by DA-6, we collected leaves (source), stems (transport), and pods (sink) at two key developmental stages. This multi-tissue approach enables a holistic understanding of how DA-6 coordinates source-sink-transport relationships to enhance yield and quality.

For each treatment and replication, five representative soybean plants were selected at the specified time points. From these plants, the third fully expanded trifoliate leaves (counted from the apex), middle stem segments (between the 3rd and 4th nodes), and young pods (approximately 1–2 cm in length at R2; developing seeds at R6) were collected. All transcriptome sequencing was performed on samples collected from the Yangluo (YL) experimental site. Tissues from the five plants per replicate were immediately pooled, rapidly frozen in liquid nitrogen, and stored at -80 °C for subsequent RNA extraction and transcriptome sequencing.

Total RNA was extracted from approximately 100 mg of ground tissue using the TRIzol reagent (Invitrogen, USA) according to the manufacturer’s protocol. RNA purity and concentration were evaluated using the NanoDrop 2000 spectrophotometer (Thermo Scientific, USA). RNA integrity was rigorously assessed using the Agilent 2100 Bioanalyzer with the RNA Nano 6000 Assay Kit (Agilent Technologies, USA). Only RNA samples with an OD 260/280 ratio between 1.8 and 2.1, an OD 260/230 ratio greater than 2.0, and an RNA Integrity Number (RIN) greater than 7.0 were used for library construction. Sequencing libraries were constructed using the TruSeq Stranded mRNA LT Sample Prep Kit (Illumina, San Diego, CA, USA) according to the manufacturer’s instructions, which included steps for mRNA enrichment, fragmentation, cDNA synthesis, adapter ligation, and PCR amplification. The transcriptome sequencing and subsequent bioinformatic analysis were conducted by OE Biotech Co., Ltd. (Shanghai, China).

### 2.5 RNA sequencing and bioinformatics analysis

The raw sequencing data (raw reads) were initially processed using Trimmomatic (v0.36) to remove reads containing adapter sequences, poly-N sequences, and low-quality bases (Bolger et al., 2014). The resulting high-quality clean reads were then mapped to the soybean reference genome (Williams 82.a2.v1) using the splice-aware aligner HISAT2 (v2.0.5) (Kim et al., 2015). The mapped reads for each gene were counted using HTSeq (v0.6.1) (Anders et al., 2015). DEGs were identified using the DESeq (Anders and Huber, 2012) R package functions estimateSizeFactors and nbinomTest. Pvalue < 0.05 and foldChange >2 or foldChange < 0.5 was set as the threshold for significantly differential expression. Hierarchical cluster analysis of DEGs was performed to explore genes expression pattern. GO enrichment and KEGG (Kanehisa et al., 2008) pathway enrichment analysis of DEGs were respectively performed using R based on the hypergeometric distribution.

### 2.6 Quantitative real-time PCR analysis

Total RNA was extracted from soybean leaves using TRIzol reagent (Invitrogen) and treated with DNase I to remove genomic DNA, followed by cDNA synthesis using HiScript^®^ II Q RT SuperMix (+gDNA wiper) kit (Vazyme). qRT-PCR was performed in triplicate for each sample on a CFX Connect Real-Time System (Bio-Rad) using SYBR Green, with cycling conditions of 45 cycles at 95 °C for 10 s and 60 °C for 30 s, and fluorescence detection at each cycle end. β-actin served as the internal reference gene for normalization, and relative gene expression levels were calculated using the 2−ΔΔCt method, with three biological replicates (each pooled from ≥5 individual plants) analyzed per tissue type.

A comprehensive **Supplementary Table** (**Supplementary Table 6**) listing all DEGs for each comparison, including Gene ID, annotation, Log_2_ fold change, p−value, adjusted p−value (FDR), and regulation status, is provided.

## 3 Results

### 3.1 DA-6 application enhances soybean yield while maintaining seed protein and oil content

Field trials at two independent locations (Yangluo, YL; Jingmen, JM) showed that foliar application of DA-6 at R1 and R5 stages significantly increased grain yield by 21.1% at YL and 11.5% at JM compared to the control (**Table 1**). Seed protein and oil contents remained stable at both locations, with no statistically significant differences between T1 and T0 treatments (**Table 1**).

**Table 1:** Effects of foliar application of DA-6 on soybean grain yield and seed quality.

Trait	Treatment	Yangluo site (YL)	Jingmen site (JM)	Pooled analysis/effect size
Grain Yield (kg ha^−1^)	T0 (Control)	3788.80 ± 98.89 a	3406.12 ± 157.20 a	—
	T1 (DA-6)	4588.71 ± 58.88 b	3797.55 ± 62.70 b	—
	Increase	+21.1%	+11.5%	+16.3% (mean)
Seed Protein Content (%)	T0 (Control)	45.70 ± 0.10 a	45.60 ± 0.70 a	45.65 ± 0.40 a
	T1 (DA-6)	46.10 ± 0.00 a	45.10 ± 0.30 a	45.60 ± 0.50 a
	Difference	+0.40 p.p.	-0.50 p.p.	-0.05 p.p.
Seed Oil Content (%)	T0 (Control)	22.50 ± 0.30 a	22.60 ± 0.40 a	22.55 ± 0.35 a
	T1 (DA-6)	22.60 ± 0.30 a	22.90 ± 0.20 a	22.75 ± 0.25 a
	Difference	+0.10 p.p.	+0.30 p.p.	+0.20 p.p.

Data are presented as mean ± SE (n = 3). Different letters within the same site and trait indicate significant differences according to LSD test at p < 0.05. p.p. = percentage points. Pooled analysis values represent the mean ± pooled SE of data from both sites (to show overall trends; significance is based on site-specific statistical tests). The percentage increase was calculated as: [(T1 mean - T0 mean)/T0 mean] × 100%.

Analysis of yield components and agronomic traits revealed that DA-6 treatment did not significantly alter plant height at YL but reduced it at JM, while increasing the number of main stem nodes and productive branches, particularly at the JM site (**Table 2**). Pods per plant and grains per plant were significantly increased by DA-6 at both locations. At the JM site, pods and grains per plant increased by 29.1% and 47.6%, respectively. Seeds per pod showed modest increases under DA-6 treatment at both sites (YL: 2.0→2.2; JM: 2.0→2.4), although these changes did not reach statistical significance (**Table 2**). These data indicate that DA-6-induced yield enhancement is primarily attributed to expanded sink capacity rather than altered plant architecture or single seed weight.

**Table 2:** Effects of foliar application of DA-6 on agronomic traits and yield components in soybean.

Trait	Treatment	Yangluo site (YL)	Jingmen site (JM)
Plant Height (cm)	T0 (Control)	63.93 ± 2.00 a	68.70 ± 3.65 a
	T1 (DA-6)	63.83 ± 4.07 a	59.70 ± 3.70 b
Main Stem Nodes (no. plant^−1^)	T0 (Control)	12.97 ± 0.21 a	12.17 ± 0.29 a
	T1 (DA-6)	13.43 ± 0.06 a	13.40 ± 0.22 b
Productive Branches (no. plant^−1^)	T0 (Control)	4.97 ± 0.25 a	2.67 ± 0.34 a
	T1 (DA-6)	5.40 ± 0.10 a	3.30 ± 0.22 b
Pods per Plant (no. plant^−1^)	T0 (Control)	42.40 ± 1.93 a	28.67 ± 3.27 a
	T1 (DA-6)	46.73 ± 0.75 b	37.00 ± 0.61 b
Grains per Plant (no. plant^−1^)	T0 (Control)	84.73 ± 5.25 a	58.57 ± 4.82 a
	T1 (DA-6)	102.83 ± 3.61 b	86.47 ± 3.55 b
Number of seeds per pod	T0 (Control)	2.0 ± 0.12 a	2.0 ± 0.14 a
	T1 (DA-6)	2.2 ± 0.04 a	2.4 ± 0.08 a

Data are presented as mean ± SE (n = 3). Different letters within the same site and trait indicate significant differences according to LSD test.

### 3.2 RNA-seq data analysis reveals widespread transcriptional reprogramming

To elucidate the molecular basis for the DA-6-mediated synergy between yield and quality, we performed a comprehensive transcriptome analysis. Plants were treated with DA-6 at R1 and R5 stages, and tissues including stems, leaves, and pods were collected 14 days post-treatment for RNA sequencing. After rigorous quality control to remove adapter sequences and low-quality reads, we obtained a high-quality dataset comprising 39.13 to 50.66 million clean reads per sample (**Supplementary Table 1**). Mapping efficiency was robust, with 37.64 to 49.12 million reads successfully aligned to the soybean reference genome.

**Figure 1:**
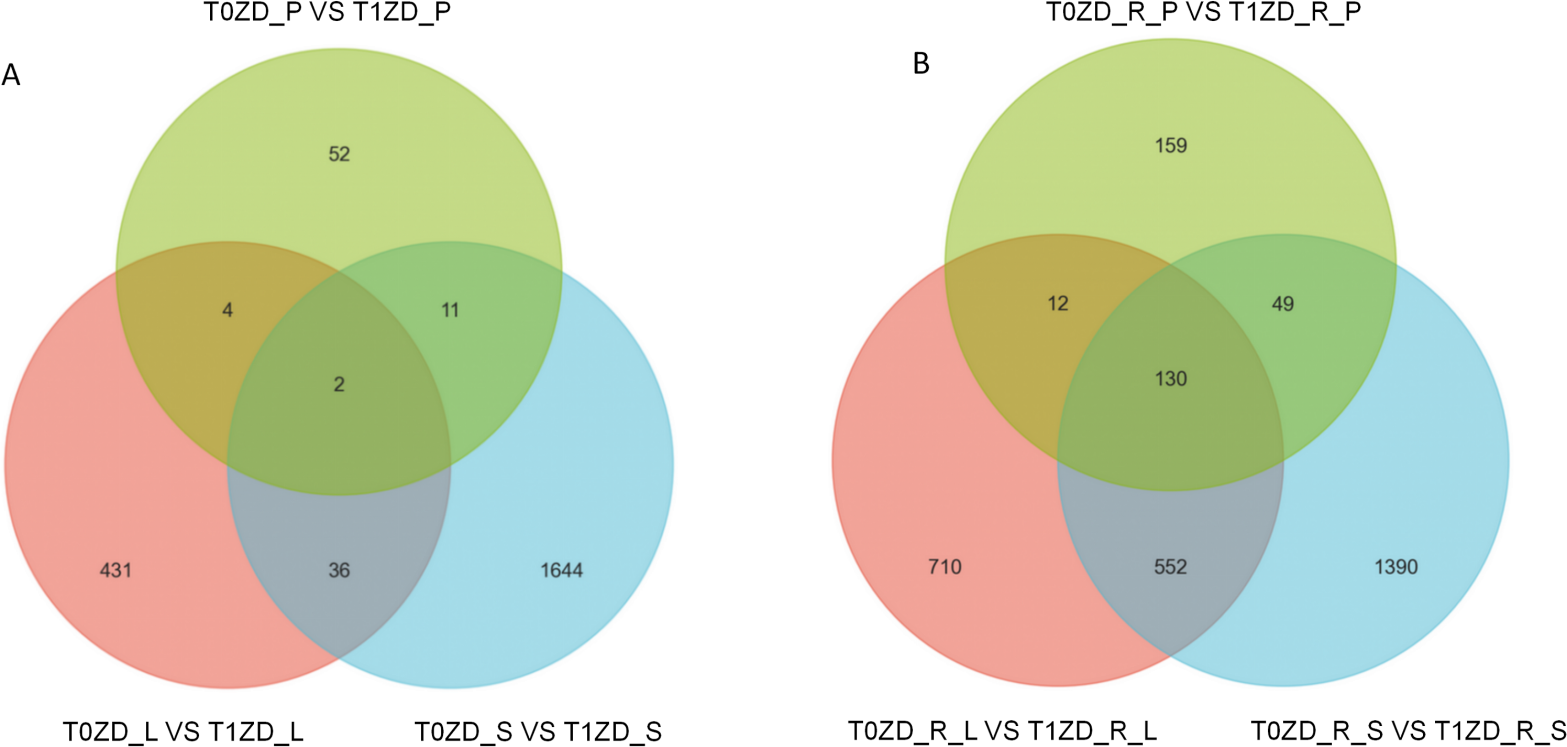
Venn diagram of differentially expressed genes (DEGs) in response to DA−6 treatment. **(A)** Flowering stage: DEGs identified in leaf (T0ZD_L vs T1ZD_L), stem (T0ZD_S vs T1ZD_S), and pod (T0ZD_P vs T1ZD_P) tissues. **(B)** Pod−filling stage: DEGs identified in leaf (T0ZD_R_L vs T1ZD_R_L), stem (T0ZD_R_S vs T1ZD_R_S), and pod (T0ZD_R_P vs T1ZD_R_P) tissues.Numbers in each region indicate the count of DEGs. Overlapping regions represent genes differentially expressed in multiple tissues.

Differential expression analysis revealed distinct tissue- and stage-specific transcriptional responses to DA-6. At the flowering stage, we identified 473 DEGs in leaves (T0ZD_L vs T1ZD_L), 1,693 in stems (T0ZD_S vs T1ZD_S), and 69 in pods (T0ZD_P vs T1ZD_P), with substantial overlap between stems and leaves (**Figure 1A**). At the pod-filling stage, the transcriptional response intensified markedly: 350 DEGs in pods (T0ZD_R_P vs T1ZD_R_P), 1,404 in leaves (T0ZD_R_L vs T1ZD_R_L), and 2,121 in stems (T0ZD_R_S vs T1ZD_R_S) (**Figure 1B**). A complete list of all DEGs, including gene annotations, expression levels, and statistical metrics, is provided in **Supplementary Table 6**.

### 3.3 Functional characterization of DEGs highlights stage-specific shifts

**Figure 2:**
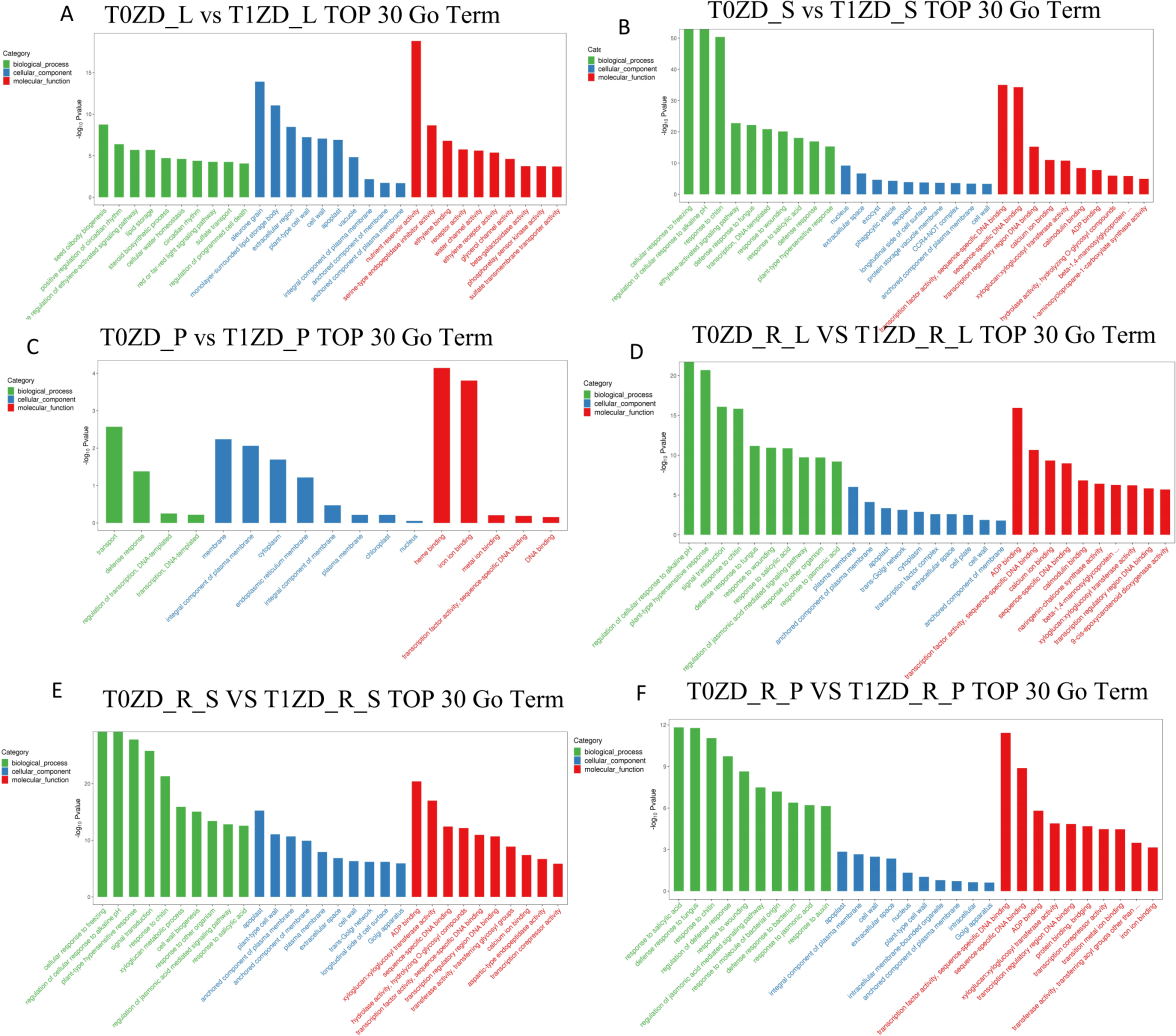
Gene Ontology (GO) enrichment analysis of DEGs across tissues and stages. The top 30 enriched GO terms (Biological Process, Cellular Component, Molecular Function) are shown for each comparison. **(A)** Flowering stage leaves (T0ZD_L vs T1ZD_L); **(B)** Flowering stage stems (T0ZD_S vs T1ZD_S); **(C)** Flowering stage pods (T0ZD_P vs T1ZD_P); **(D)** Pod-filling stage leaves (T0ZD_R_L vs T1ZD_R_L); **(E)** Pod-filling stage stems (T0ZD_R_S vs T1ZD_R_S); **(F)** Pod-filling stage pods (T0ZD_R_P vs T1ZD_R_P). Terms are ranked by enrichment significance (−log_10_(p-value)). Bar color indicates GO category.

GO enrichment analysis was performed across all tissue-stage comparisons (**Figure 2**; **Supplementary Table 2**).

At the flowering stage, leaves exhibited enrichment of terms associated with seed oil body biogenesis (GO:0010344) and nutrient reservoir activity (GO:0045735) (**Figure 2A**). Stems showed enrichment of terms related to cellular response to alkaline pH and plasma membrane (**Figure 2B**). Pods displayed limited GO enrichment (**Figure 2C**).

During the pod-filling stage, leaves showed enrichment in ADP binding and regulation of cellular response to alkaline pH (**Figure 2D**). Stems exhibited enrichment in plasma membrane and carbohydrate metabolic process (**Figure 2E**). Pods were enriched for protein folding and response to oxidative stress (**Figure 2F**).

**Figure 3:**
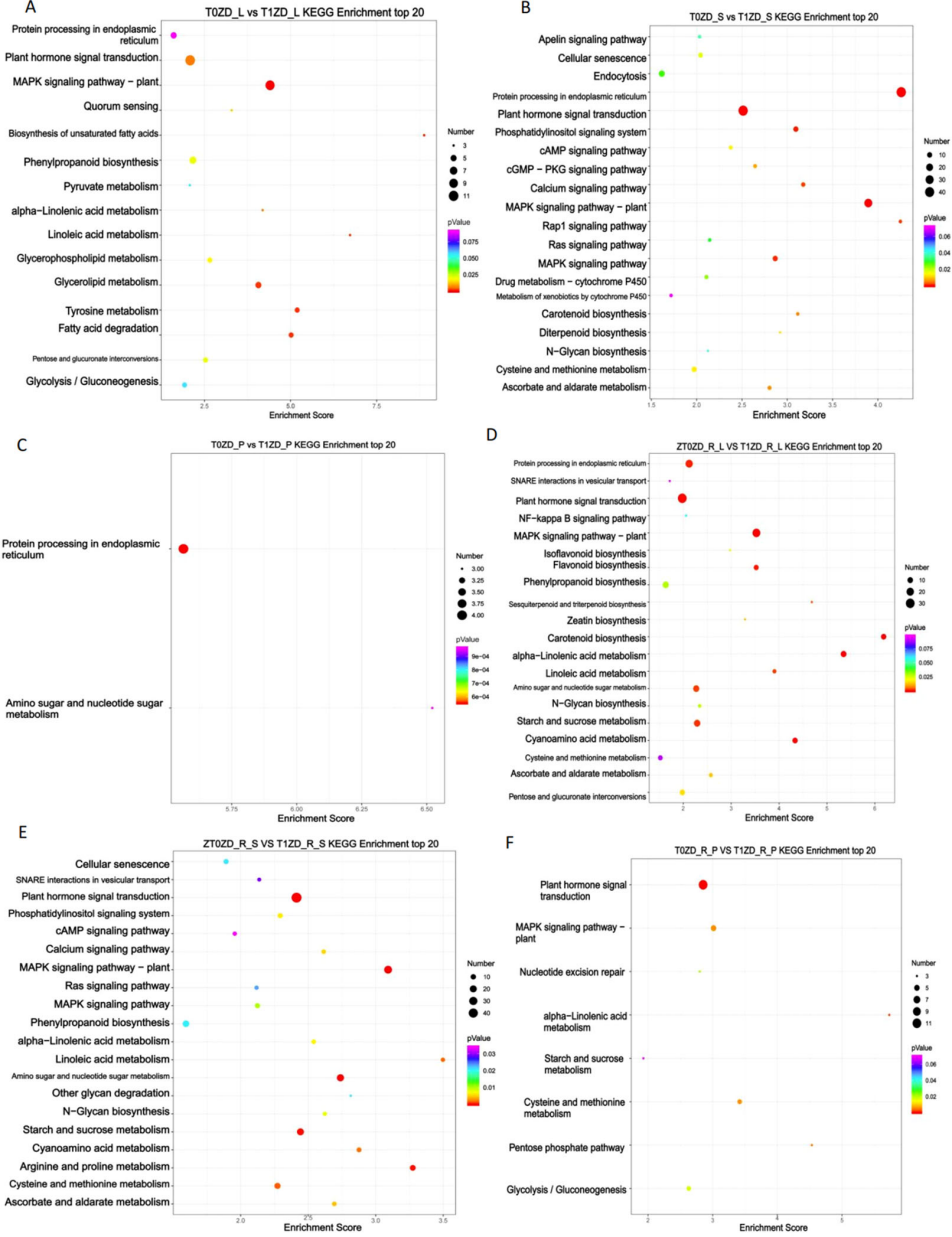
KEGG pathway enrichment analysis of DEGs across tissues and developmental stages. The top enriched KEGG pathways are shown for each comparison (ranked by enrichment significance). **(A)** Flowering stage leaves (T0ZD_L vs T1ZD_L). **(B)** Flowering stage stems (T0ZD_S vs T1ZD_S). **(C)** Flowering stage pods (T0ZD_P vs T1ZD_P). **(D)** Pod-filling stage leaves (T0ZD_R_L vs T1ZD_R_L). **(E)** Pod-filling stage stems (T0ZD_R_S vs T1ZD_R_S). **(F)** Pod-filling stage pods (T0ZD_R_P vs T1ZD_R_P). Pathway names are listed along the y-axis; enrichment score (−log_10_(p-value)) is shown on the x-axis.

KEGG pathway enrichment analysis (**Figure 3**; **Supplementary Table 3**) showed that at flowering, leaves were enriched in protein processing in endoplasmic reticulum, plant hormone signal transduction, and MAPK signaling (**Figure 3A**). Stems showed enrichment in endocytosis, plant hormone signal transduction, and multiple signaling cascades (**Figure 3B**). Pods exhibited minimal pathway changes (**Figure 3C**). During pod-filling, leaves were enriched in starch and sucrose metabolism, plant hormone signal transduction, and α-linolenic acid metabolism (**Figure 3D**). Stems showed enrichment in MAPK signaling, amino sugar and nucleotide sugar metabolism, starch and sucrose metabolism, and α-linolenic acid metabolism (**Figure 3E**). Pods were enriched for isoflavonoid biosynthesis, flavonoid biosynthesis, and plant hormone signal transduction (**Figure 3F**).

### 3.4 DA-6 orchestrates spatiotemporal reprogramming of carbohydrate metabolism

**Figure 4:**
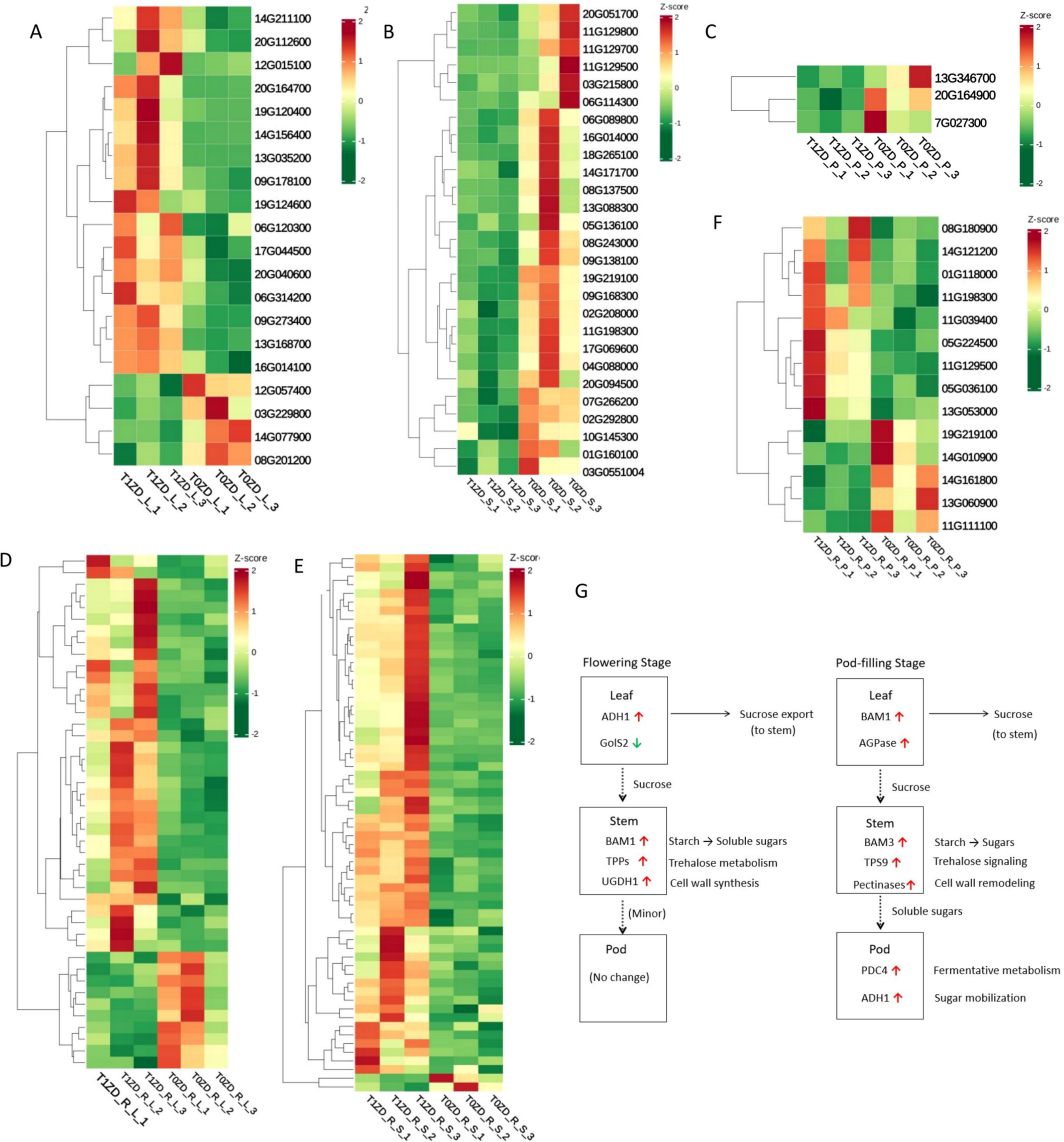
Heatmap of DEGs involved in carbohydrate metabolism across tissues and developmental stages. Each row represents a gene, each column represents a biological replicate. Expression values are Z−score normalized (red: up−regulation, green: down−regulation). **(A)** Flowering stage leaves (T0ZD_L vs T1ZD_L). **(B)** Flowering stage stems (T0ZD_S vs T1ZD_S). **(C)** Flowering stage pods (T0ZD_P vs T1ZD_P). **(D)** Pod-filling stage leaves (T0ZD_R_L vs T1ZD_R_L). **(E)** Pod-filling stage stems (T0ZD_R_S vs T1ZD_R_S). **(F)** Pod-filling stage pods (T0ZD_R_P vs T1ZD_R_P). Selected key genes are annotated on the right. **(G)** Schematic model of DA-6-mediated carbohydrate metabolic reprogramming. Key enzymatic steps and transcriptional changes are summarized across leaves, stems, and pods at both developmental stages. Red arrows/genes: upregulation; green: downregulation; black: no change/not detected.

DEGs involved in carbohydrate metabolism were identified using KEGG carbohydrate metabolism pathways (map00010–map00062) and GO terms including carbohydrate metabolic process (GO:0005975), starch metabolism (GO:0005982), and trehalose metabolism (GO:0005991). Expression dynamics are presented in **Figure 4**; **Supplementary Table 4**.

At the flowering stage, leaves showed upregulation of alcohol dehydrogenases (ADH1, ADH-like 1) and aldehyde dehydrogenase (ALDH3F1), and downregulation of galactinol synthase 2 (GolS2) (**Figure 4A**). Stems showed upregulation of beta-amylase 1 (BAM1), trehalose-phosphate phosphatases (TPP J, I, 4), UDP-glucose 6-dehydrogenase (UGDH1), and UDP-glucuronate 4-epimerase (GAE1) (**Figure 4B**). Pods exhibited limited transcriptional changes (**Figure 4C**).

At the pod-filling stage, leaves showed sustained upregulation of beta-glucosidases, BAM1, hexokinase-4 (HXK4), and ADP-glucose pyrophosphorylase (AGPase) (**Figure 4D**). Stems showed upregulation of genes involved in starch degradation (BAM3, beta-glucosidases), trehalose metabolism (TPPs, TPS9), cell wall modification, and glycolysis (**Figure 4E**). Pods showed upregulation of pyruvate decarboxylase 4 (PDC4), ADH1, BAM3, and TPS9 (**Figure 4F**).

A schematic summary of these metabolic changes is presented in **Figure 4G**.

### 3.5 DA-6 modulates the expression of photosynthesis-related genes

To determine whether DA-6 enhances yield through transcriptional regulation of photosynthetic capacity, we analyzed differentially expressed genes associated with photosynthesis. Photosynthesis-related DEGs were identified using GO terms for photosynthesis (GO:0015979), photosystem I/II (GO:0009522, GO:0009523), light harvesting (GO:0009765), and chloroplast function (GO:0009507), as well as KEGG pathway map00195 (photosynthesis).

**Figure 5:**
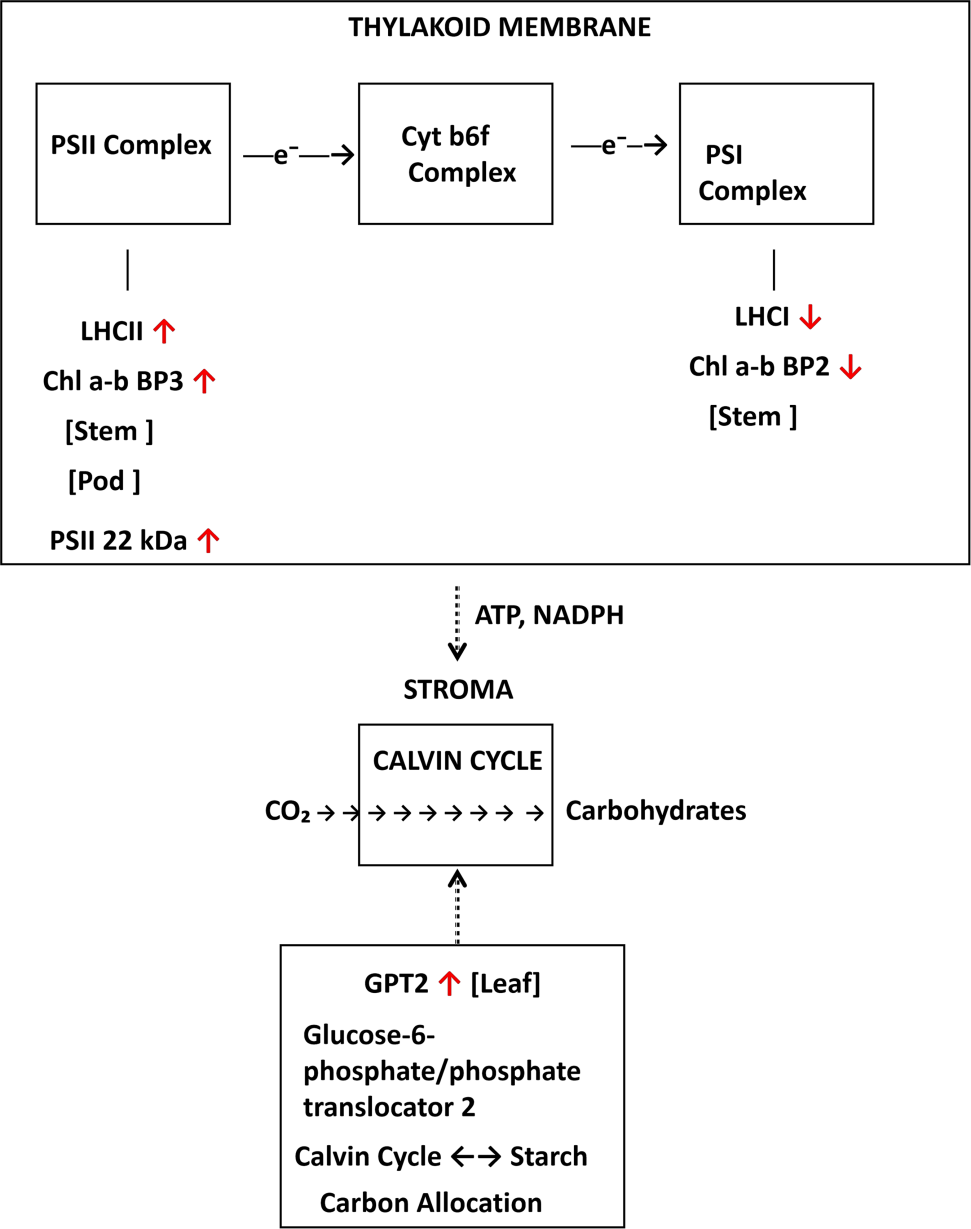
Schematic model of DA-6-mediated involved in photosynthesis across tissues and developmental stages. Key enzymatic steps and transcriptional changes are summarized across leaves, stems, and pods at both developmental stages. Red arrows/genes: upregulation; green: downregulation; black: no change/not detected.

DA-6 induced tissue-specific and stage-dependent transcriptional changes in photosynthetic components, with effects markedly amplified during pod-filling (**Figure 5**; **Table 3**).

**Table 3:** The expression of photosynthesis-related genes.

Gene ID	Description	Regulation
T0ZD_L VS T1ZD_L		
Glyma.06G125700	photosystem II assembly	down
T0ZD_P VS T1ZD_P		
Glyma.16G165200	Chlorophyll a-b binding protein	up
T0ZD_S VS T1ZD_S		
Glyma.06G113200	Photosystem II 22 kDa protein	up
T0ZD_R_L VS T1ZD_R_L		
Glyma.06G045400	Zinc finger protein ZAT10;photosynthesis;response to high light intensity	up
Glyma.13G206700	Glucose-6-phosphate/phosphate translocator 2;regulation of photosynthesis	up
Glyma.14G088300	Zinc finger protein ZAT10;photosynthesis	up
Glyma.02G015800	Fumarate hydratase 2	up
T0ZD_R_P VS T1ZD_R_P		
Glyma.04G249700	Photosystem II 22 kDa protein	up
T0ZD_R_S VS T1ZD_R_S		
Glyma.05G128000	Chlorophyll a-b binding protein 3	up
Glyma.14G088300	Zinc finger protein ZAT10;photosynthesis	up
Glyma.17G236200	Zinc finger protein ZAT10;photosynthesis	up
Glyma.19G261400	Photosystem I chlorophyll a/b-binding protein 2	down

At the flowering stage, regulatory changes were limited. In leaves, SigE—encoding a sigma factor involved in chloroplast development—was downregulated. In stems, Photosystem II 22 kDa protein was upregulated.

During pod-filling, a broader and more pronounced activation was observed. As illustrated in **Figure 5**, multiple components of the photosynthetic apparatus were differentially regulated in a tissue-specific manner. In leaves, two copies of ZAT10 and Glucose-6-phosphate/phosphate translocator 2 (GPT2) were significantly upregulated.

This positive regulation extended to sink tissues. In pods, Photosystem II 22 kDa protein was upregulated. Stems exhibited coordinated upregulation of light-harvesting complex II components (Chlorophyll a-b binding protein 3, LHCII) and two additional ZAT10 copies, coupled with downregulation of Photosystem I chlorophyll a/b-binding protein 2 (LHCI).

### 3.6 DA-6 elicits a ‘seed-like’ transcriptional program in leaf tissues

**Figure 6:**
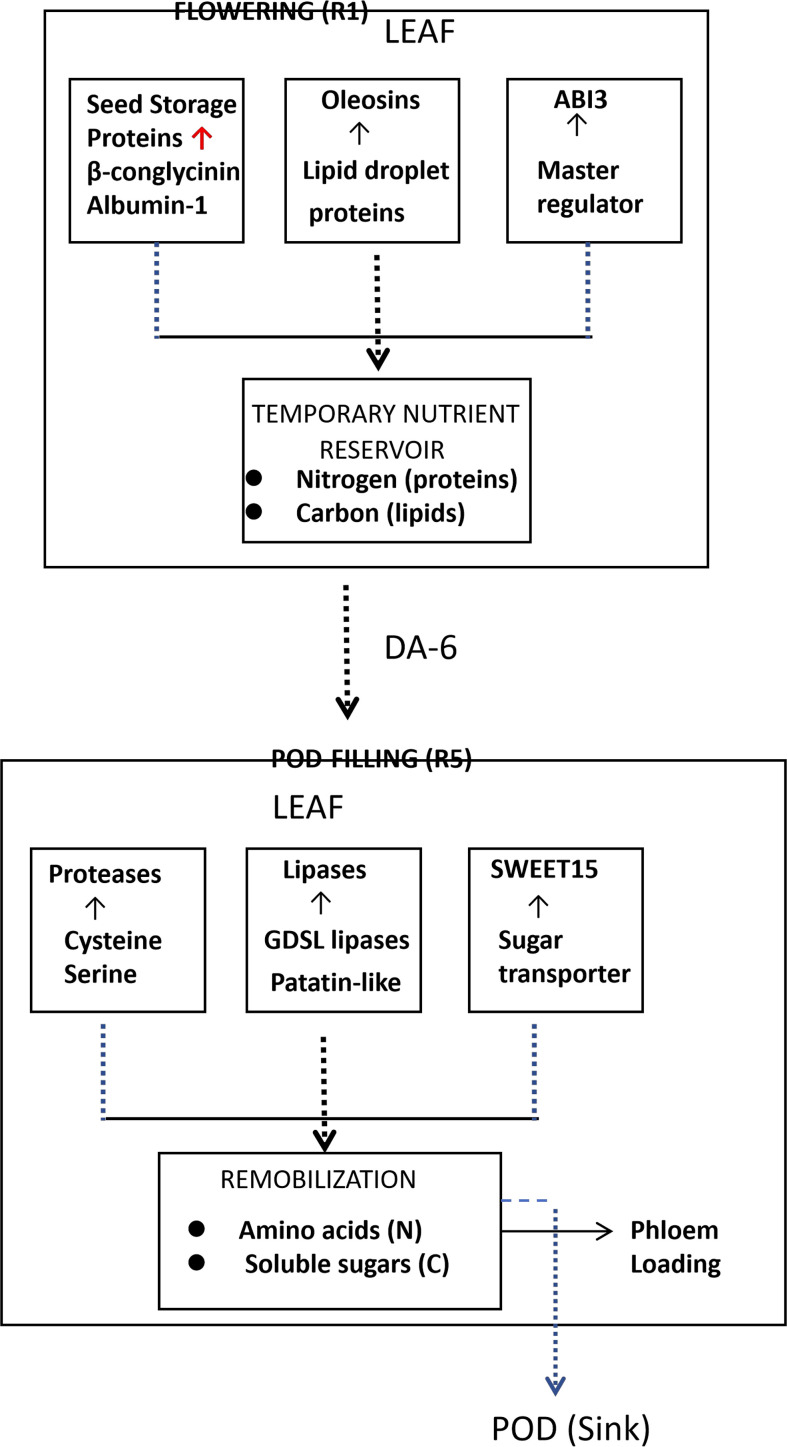
Schematic model of DA-6 activates a seed-specific transcriptional program in soybean leaves. Key enzymatic steps and transcriptional changes are summarized across leaves, stems, and pods at both developmental stages. Red arrows/genes: upregulation; green: downregulation; black: no change/not detected.

To uncover the comprehensive mechanisms by which DA-6 coordinates source-sink relationships, we identified genes that were silent in control leaves but significantly induced upon DA-6 treatment. Specifically, we screened for genes with TPM = 0 in all three replicates of T0ZD_L (control leaves) and TPM > 5 in at least two replicates of T1ZD_L (DA-6-treated leaves). These were further annotated using the SoyBase and NCBI databases to identify those canonically associated with seed development, including seed storage proteins, oleosins, late embryogenesis abundant (LEA) proteins, and master regulators of seed maturation (e.g., ABI3). This analysis revealed a profound transcriptional reprogramming, wherein DA-6 activated a suite of genes that are typically expressed specifically during seed development (**Table 4**; **Figure 6**). These included:

**Table 4:** Representative genes activated *de novo* in leaves by DA-6 treatment.

Gene ID	Description	Putative function in leaves
Glyma.20G148400	Beta-conglycinin, alpha chain	Temporary Nitrogen Storage
Glyma.13G194400	Albumin-1 chain a	Temporary Nitrogen Storage
Glyma.10G193900	Oleosin 1	Temporary Carbon/Lipid Storage
Glyma.08G357600	B3 domain-containing transcription factor ABI3	Activation of Seed Storage Program
Glyma.U018200	Late embryogenesis abundant protein 3	Stress Protection & Homeostasis
Glyma.09G158800	Bowman-Birk type proteinase inhibitor C-II	Protection of Protein Reserves
Glyma.08G183500	Bidirectional sugar transporter SWEET15	Sugar Transport for Remobilization

Major seed storage proteins: Genes encoding the alpha chain of β-conglycinin (Glyma.20G148300, Glyma.20G148400) and albumin-1 (Glyma.13G194400).Oil body-associated proteins: Multiple oleosin genes (Glyma.10G193900, Glyma.17G086400, Glyma.20G196600), which are crucial for the formation of lipid storage organelles. Master regulators of seed maturation: Transcription factors such as ABI3 (Glyma.08G357600, Glyma.18G176100). Late embryogenesis and desiccation-related proteins: LEA protein (Glyma.U018200) and desiccation-related proteins. Defense and protease inhibitors: Defensin-like protein, Snakin-2, and Bowman-Birk type protease inhibitors.

### 3.7 DA-6 reinforces and temporally regulates plant hormone signal transduction

**Figure 7:**
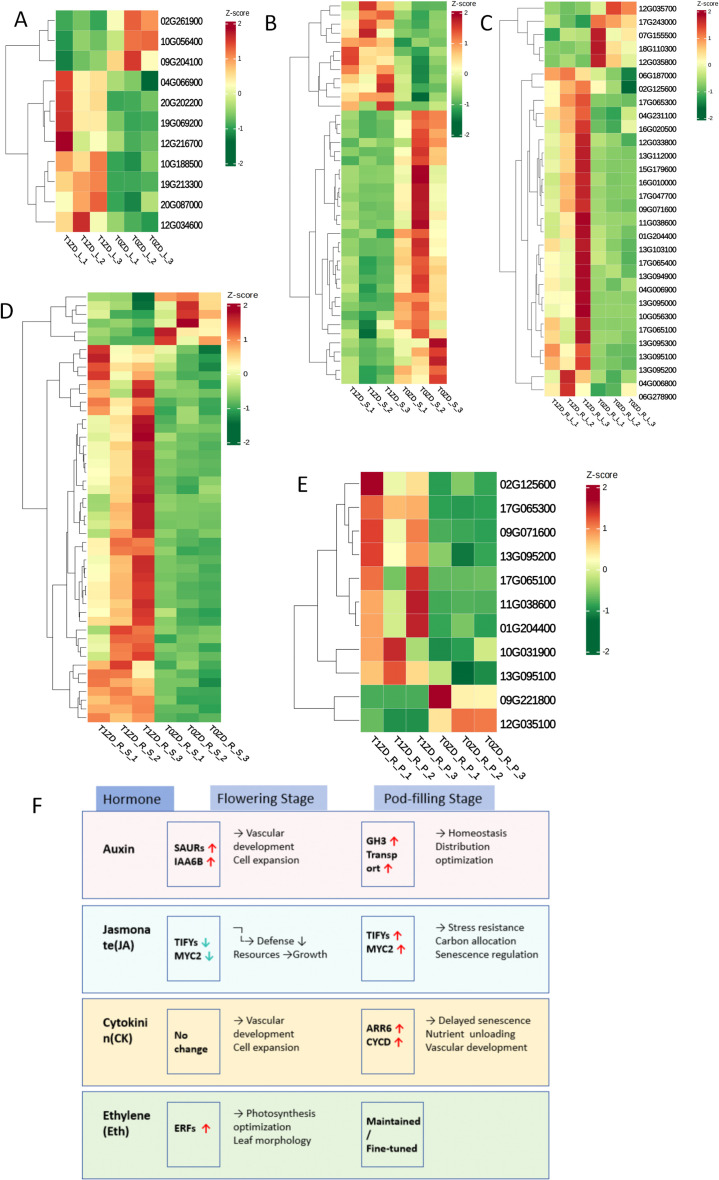
Heatmap of DEGs involved in plant hormone signal transduction across tissues and developmental stages. Each row represents a hormone−related gene, each column represents a biological replicate. Expression values are Z−score normalized (red: up−regulation, green: down−regulation). **(A)** Flowering stage leaves (T0ZD_L vs T1ZD_L). **(B)** Flowering stage stems (T0ZD_S vs T1ZD_S). **(C)** Pod-filling stage leaves (T0ZD_R_L vs T1ZD_R_L). **(D)** Pod-filling stage stems (T0ZD_R_S vs T1ZD_R_S). **(E)** Pod-filling stage pods (T0ZD_R_P vs T1ZD_R_P). Hormone pathways are indicated on the right (auxin, jasmonate, cytokinin, ethylene, etc.). The flowering-stage pod comparison (T0ZD_P vs T1ZD_P) is omitted due to insufficient hormone-related DEGs. **(F)** Schematic model of DA-6-mediated plant hormone signal transduction. Key enzymatic steps and transcriptional changes are summarized across leaves, stems, and pods at both developmental stages. Red arrows/genes: upregulation; green: downregulation; black: no change/not detected.

Our transcriptomic analysis reveals that DA-6 orchestrates temporal regulation of hormone signaling pathways (**Figure 7**). A schematic pathway map summarizing key DEGs in hormone signaling is provided in **Figure 7F**. Hormone-related DEGs were retrieved by mapping all DEGs to the KEGG plant hormone signal transduction pathway (map04075) and to GO terms associated with hormone-mediated signaling pathways (GO:0009755 for auxin, GO:0009753 for jasmonic acid, GO:0009736 for cytokinin, GO:0009723 for ethylene, etc.).

During the flowering stage, DA-6 induced distinct tissue-specific hormonal responses. In stems, auxin-responsive genes (e.g., SAUR20, SAUR23, IAA6B) were upregulated, whereas jasmonate signaling components (e.g., TIFY transcription factors and MYC2) were downregulated (**Figure 7B**). Leaves exhibited elevated expression of ethylene signaling elements (**Figure 7A**).

During the pod-filling stage, jasmonate signaling genes were upregulated across all examined tissues (**Figures 7C–E**). This activation was accompanied by upregulation of cytokinin response regulators (e.g., ARR6) in stems. Stem tissues also showed increased expression of D-type cyclins (**Figure 7D**). Auxin homeostasis-related genes, including GH3 conjugating enzymes and auxin transporters, were upregulated in stems (**Figure 7D**).

### 3.8 Stage- and tissue-specific transcription factor networks respond to DA-6

**Figure 8:**
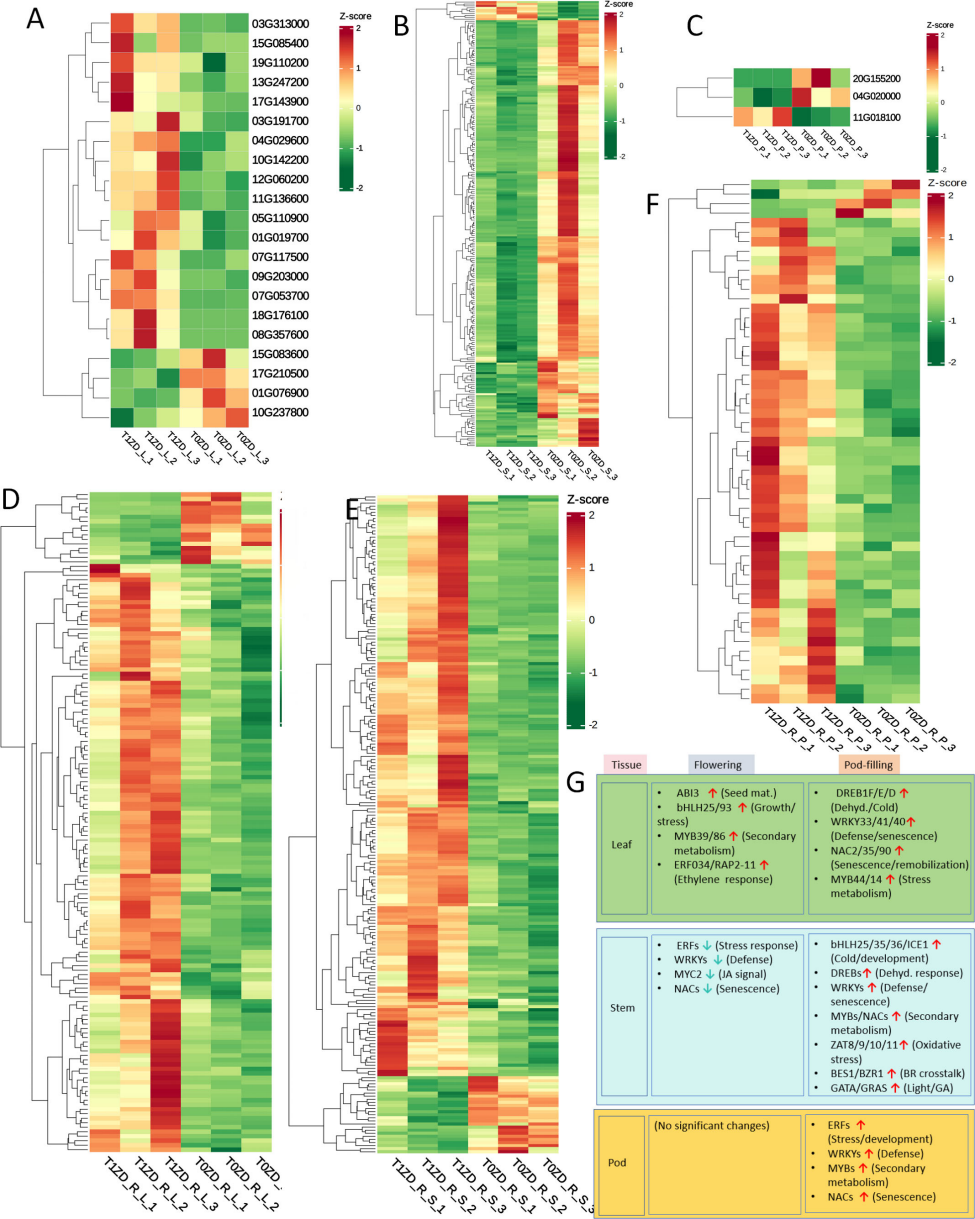
Heatmap of differentially expressed transcription factors (TFs) across tissues and developmental stages. Each row represents a TF gene, each column represents a biological replicate. Expression values are Z−score normalized (red: up−regulation, green: down−regulation). **(A)** Flowering stage leaves (T0ZD_L vs T1ZD_L). **(B)** Flowering stage stems (T0ZD_S vs T1ZD_S). **(C)** Flowering stage pods (T0ZD_P vs T1ZD_P). **(D)** Pod-filling stage leaves (T0ZD_R_L vs T1ZD_R_L). **(E)** Pod-filling stage stems (T0ZD_R_S vs T1ZD_R_S). **(F)** Pod-filling stage pods (T0ZD_R_P vs T1ZD_R_P). **(G)** Schematic model of DA-6-mediated TFs. Key enzymatic steps and transcriptional changes are summarized across leaves, stems, and pods at both developmental stages. Red arrows/genes: upregulation; green: downregulation; black: no change/not detected.

To identify transcriptional regulators mediating DA-6’s pleiotropic effects, we analyzed differentially expressed transcription factors across tissues and developmental stages (**Figures 8A–F**; **Supplementary Table 5**). A regulatory network diagram is summarized in **Figure 8G**. TF-encoding DEGs were identified by screening all DEGs against the Plant Transcription Factor Database and further classified by family (e.g., ERF, WRKY, MYB, NAC, bHLH, B3).

During the flowering stage, DA-6 induced distinct tissue-specific TF expression patterns. In leaves (**Figure 8A**), two B3 domain-containing transcription factors (ABI3) were upregulated, along with bHLH25, bHLH93, MYB39, MYB86, and ethylene-responsive TFs (RAP2-11, ERF034). In stems (**Figure 8B**), downregulation was observed across multiple TF families (ERF, WRKY, bHLH, MYB, NAC), including the jasmonate signaling component MYC2, stress-responsive ERFs, and defense-related WRKYs. TF changes in pods at this stage were minimal (**Figure 8C**).

During the pod-filling stage, DA-6 reversed the earlier suppression pattern and triggered widespread activation of TF networks. In leaves (**Figure 8D**), upregulation of dehydration-responsive ERFs (DREB1F, DREB1E, DREB1D), WRKYs (WRKY33, WRKY41, WRKY40), MYBs (MYB44, MYB14), and NACs (NAC2, NAC35, NAC90) was observed. The most extensive TF reprogramming was detected in stems (**Figure 8E**), with upregulation of bHLHs (bHLH25, bHLH35, bHLH36, ICE1), dehydration-responsive ERFs, defense/senescence-regulating WRKYs, secondary metabolism-related MYBs and NACs, and zinc finger proteins (ZAT8, ZAT9, ZAT10, ZAT11). Additionally, brassinosteroid signaling regulator BES1/BZR1 homolog protein 4, GATA TFs, and GRAS family proteins were upregulated in stems. In pods (**Figure 8F**), upregulation of ERF, WRKY, MYB, and NAC members was observed.

### 3.9 WGCNA identifies core gene modules associated with DA-6-induced synergy

**Figure 9:**
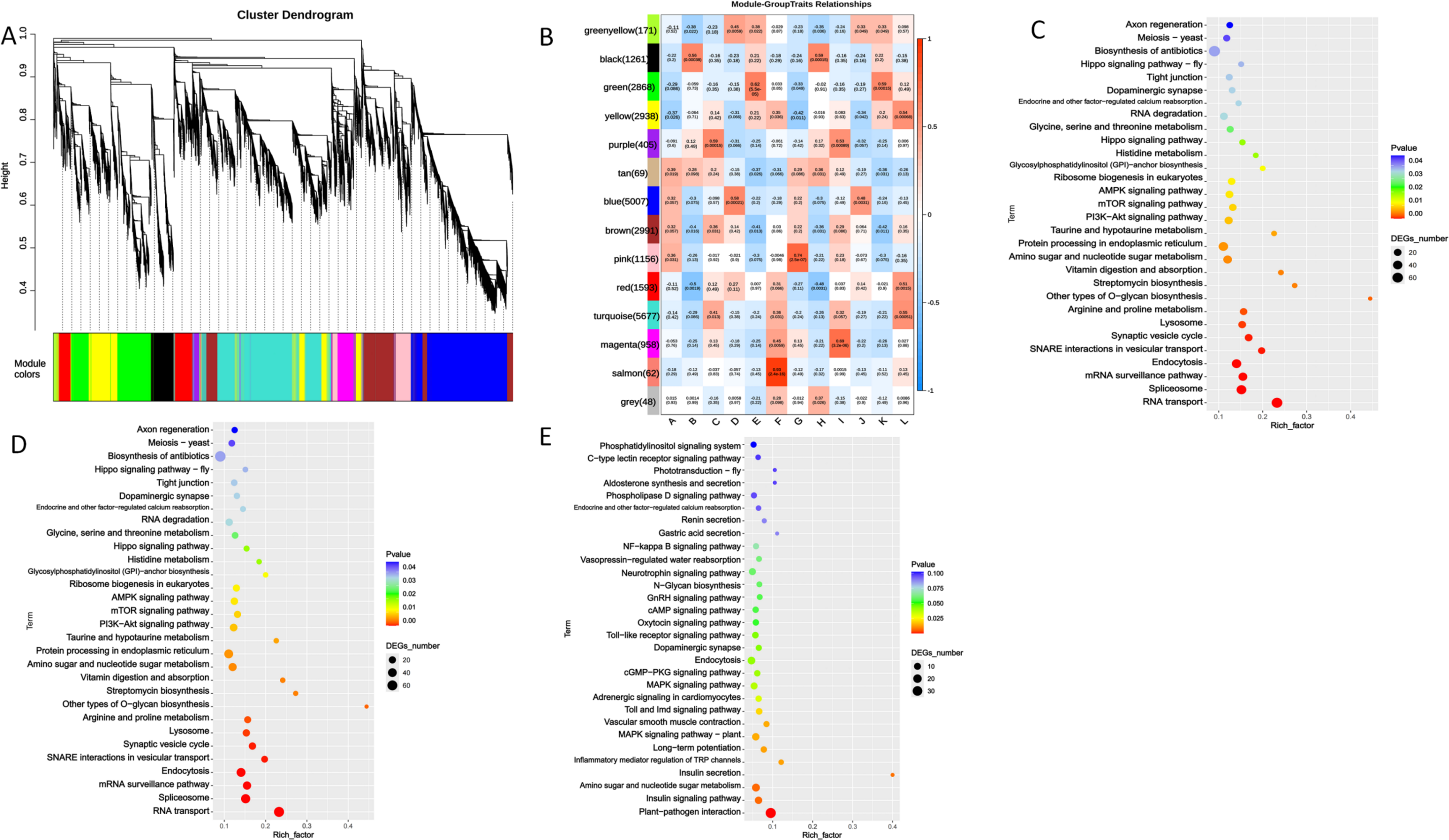
Weighted gene co-expression network analysis of DEGs. **(A)** Clustering dendrograms of protein and module division, with dissimilarity based on the topological overlap, together with assigned module colors. Overall, 13 co-expression modules were constructed and are shown in different colors. **(B)** Module-sample group association analysis. Each row corresponds to a module, labelled with color as in panel. **(C)** The yellow module of Ko.enrich. **(D)** The pink module of Ko.enrich. **(E)** The magenta module of Ko.enrich.

To systematically identify core gene modules and regulatory networks associated with DA-6-mediated yield and quality enhancement, we performed weighted gene co-expression network analysis using transcriptome data from all collected samples. This analysis clustered 25,205 variably expressed genes into 14 distinct co-expression modules, each labeled with a unique color (**Figure 9A**).

Correlation analysis between each module’s eigengene and key experimental traits (treatment, tissue, developmental stage) was calculated (**Figure 9B**). Three modules showed high association with DA-6 application:

The Yellow Module exhibited a significant positive correlation with DA-6 treatment specifically in stems at the flowering stage (T1ZD_S). KEGG enrichment analysis showed enrichment in ‘Ribosome biogenesis in eukaryotes’, ‘AMPK signaling pathway’, ‘mTOR signaling pathway’, and ‘Amino sugar and nucleotide sugar metabolism’ (**Figure 9C**).

The Pink and Magenta Modules were strongly correlated with DA-6 treatment during the pod-filling stage in different organs. The pink module was positively correlated with DA-6 treatment in leaves at the pod-filling stage (T1ZD_R_L) and was enriched for ‘MAPK signaling pathway - plant’ and ‘Plant-pathogen interaction’ (**Figure 9D**). The magenta module showed the strongest positive correlation with DA-6 treatment in stems at the pod-filling stage (T1ZD_R_S) and with final yield, and was enriched for ‘MAPK signaling pathway - plant’, ‘Amino sugar and nucleotide sugar metabolism’, and ‘Endocytosis’ (**Figure 9E**).

To establish statistical links between transcriptional reprogramming and agronomic performance, we performed correlation analyses between expression levels of DA-6-induced metabolic genes and yield components. Expression of Beta-amylase 3 (BAM3, Glyma.01G203400) in stems at the R6 stage showed positive correlation with grains per plant (r = 0.94, p < 0.01) and final grain yield (r = 0.91, p < 0.05). Expression of trehalose-phosphate synthase 9 (TPS9, Glyma.05G036100) in pods was positively correlated with grains per plant (r = 0.87, p < 0.05) (**Figure 9F**).

### 3.10 Validation of differentially accumulated proteins by qPCR

To validate the RNA-seq results, we randomly selected 21 DEGs for qRT-PCR assays. 19 DEGs in the qRT-PCR assays were essentially consistent with their transcript abundance changes identified by RNA-seq (**Table 5**), suggesting the transcriptome data were reliable. Although the expression levels of some genes are not exactly the same in magnitude, the trend of upregulation or downregulation is consistent in both the qPCR and RNA-seq data.

**Table 5:** Verification of some differentially expressed genes by qPCR.

Comparison group	Gene ID	Ratio in RNAseq	Ratio in qRT PCR
T0ZD_L VS T1ZD_L	Glyma.06G314200	5.20	1.69
	Glyma.20G112600	2.09	1.27
	Glyma.20G040600	2.18	1.59
	Glyma.08G201200	0.32	0.74
	Glyma.12G057400	0.42	0.53
T0ZD_P VS T1ZD_P	Glyma.17G027300	0.48	0.78
T0ZD_S VS T1ZD_S	Glyma.02G292800	0.34	3.77
	Glyma.06G089800	0.23	0.37
	Glyma.07G266200	0.43	0.42
	Glyma.17G069600	0.36	0.86
	Glyma.20G094500	0.43	0.53
T0ZD_R_L VS T1ZD_R_L	Glyma.02G015800	6.76	2.48
	Glyma.13G088300	6.54	1.76
	Glyma.11G095600	5.58	1.79
	Glyma.09G138100	2.39	2.78
	Glyma.08G249900	2.60	0.36
	Glyma.20G112600	0.23	0.57
T0ZD_R_S VS T1ZD_R_S	Glyma.08G137500	15.42	2.27
	Glyma.13G076900	3.83	5.07
	Glyma.18G204200	2.06	1.32
	Glyma.12G180300	2.32	1.44

## 4 Discussion

### 4.1 A temporally orchestrated hormonal symphony

Our transcriptomic data reveal that DA-6 does not uniformly activate or suppress hormone pathways; rather, it executes temporal regulation aligned with developmental priorities. At flowering, auxin-responsive genes are upregulated in stems and jasmonate signaling genes are downregulated. During pod-filling, jasmonate signaling genes are upregulated across all tissues, accompanied by cytokinin response regulator and D-type cyclin upregulation in stems.

This phased hormonal pattern—auxin activation and jasmonate suppression at flowering, followed by jasmonate and cytokinin activation at pod-filling—represents a developmental switch in hormone signaling priorities. Jasmonates are increasingly recognized as regulators of reproductive development, carbon partitioning, and senescence under non-stress conditions (Guo et al., 2018). Their activation during pod-filling in this study occurs without stress imposition, indicating recruitment of jasmonate signaling for developmental functions rather than stress responses. Cytokinin response regulator upregulation aligns with known functions in delaying senescence and promoting nutrient mobilization (Kudo et al., 2010; Anfang and Shani, 2021). D-type cyclin upregulation in stems is consistent with enhanced cell division activity that may support vascular development.

The stage-specific regulation of jasmonate signaling emerges as a central node through which DA-6 coordinates source activity, sink strength, and transport efficiency. This temporal hormone coordination provides a mechanistic framework for optimal resource allocation across reproductive development.

### 4.2 Spatiotemporal reprogramming of carbon metabolism

DA-6 induces tissue-specific and stage-specific changes in carbohydrate metabolism gene expression. The most extensive reprogramming occurs in stems during pod-filling, with upregulation of genes encoding starch degradation enzymes (BAM1/3), trehalose metabolism enzymes (TPPs, TPS9), and cell wall remodeling proteins (pectinesterases, polygalacturonase, UGDH1). This transcriptional profile indicates stem transformation from a passive conduit to an active metabolic hub that mobilizes stored carbon reserves.

The tissue-specific contrast in GolS2 expression—downregulated in leaves and upregulated in stems at flowering—reflects differential carbon partitioning priorities. In leaves, reduced GolS2 may redirect carbon from raffinose family oligosaccharides toward sucrose export. In stems, increased GolS2 may prepare for osmoprotection or temporary carbon storage (Zhang et al., 2024).

Photosynthesis-related gene expression changes show tissue-specific patterns. During pod-filling, leaves upregulate GPT2, which coordinates carbon allocation between the Calvin cycle and starch synthesis. Stems and pods upregulate Photosystem II 22 kDa protein and light-harvesting complex II components, while stems downregulate Photosystem I chlorophyll a/b-binding protein 2. This opposing regulation of LHCII and LHCI indicates photosystem rebalancing rather than global transcriptional activation.

### 4.3 Leaf ‘priming’ as a temporary nutrient reservoir: a novel mechanism for breaking the yield-quality trade-off

DA-6 induces a seed-specific transcriptional program in vegetative leaves, including *de novo* expression of genes encoding seed storage proteins (β-conglycinin, albumin), oleosins, and the seed maturation master regulator ABI3. GO enrichment of “aleurone grain” at flowering further supports priming of protein storage infrastructure in leaves.

This phenomenon is interpreted as establishment of temporary nutrient reservoirs in source leaves. Seed storage protein synthesis provides nitrogen buffering capacity; oleosin induction suggests potential for transient lipid storage. Protease inhibitor upregulation may protect these reserves from premature degradation, while sugar transporter (SWEET15) induction supports subsequent nutrient efflux.

Although GO terms related to oil body biogenesis and oleosin-encoding genes were enriched in DA-6-treated leaves, leaf oil content was not measured and seed oil content remained unchanged. Induction of oil body-associated genes in vegetative tissues does not necessarily equate to substantial triacylglycerol accumulation; oleosin expression can occur without concomitant lipid storage and may reflect preparatory states for membrane remodeling. Even if transient lipid droplets form in leaves, they are likely rapidly mobilized and re-exported as carbon skeletons during peak pod-filling demand. Seed oil content is a seed-autonomous trait determined by embryonic metabolic capacity and carbon precursor supply during filling, not by leaf transcriptional signatures alone.

Thus, the ‘seed-like’ leaf program is best interpreted as a preparatory transcriptional state that enables leaves to serve as transient nutrient buffers. This interpretation aligns with the observation that seed protein and oil content remained stable despite significant yield gains—the increased sink capacity was met with proportionally increased nitrogen and carbon delivery from primed source tissues.

**Figure 10:**
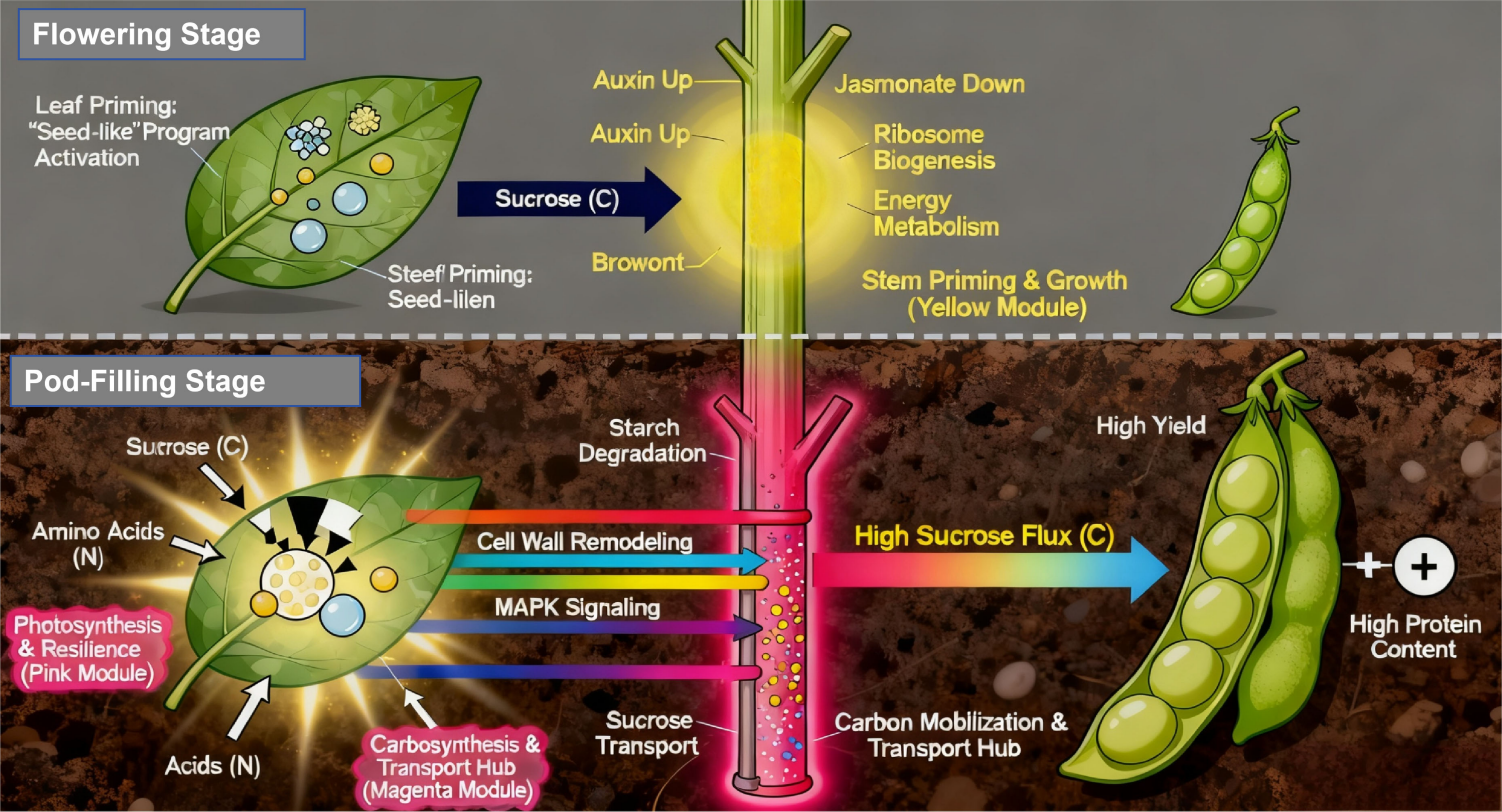
A Unified Model Illustrating the Spatiotemporal Mechanisms of DA-6 in Synergistically Enhancing Soybean Yield and Quality.

This source-leaf priming mechanism is integrated into the unified model (**Figure 10**). During flowering and early pod stages, DA-6 primes leaves as dynamic nutrient banks. During peak pod-filling demand, stored reserves are mobilized, providing sustained amino acid and carbon skeleton supply to developing seeds.

### 4.4 The central role of signaling hubs and transcriptional networks

The WGCNA results provide a systems-level validation of our findings and identify core regulatory modules (**Figure 9**). The yellow module, associated with flowering-stage stems and enriched in ribosome biogenesis and energy metabolism pathways, indicates that DA-6 enhances biosynthetic and energetic capacity during early development.

The subsequent activation of the pink module in pod-filling leaves and the magenta module in pod-filling stems, both enriched in the ‘MAPK signaling pathway’, points to a centralized signaling mechanism coordinating source and transport organs. The MAPK cascade integrates diverse developmental and environmental signals and regulates downstream cellular processes including gene expression, hormone signaling, and cytoskeleton organization (Zhang and Zhang, 2022). The co-enrichment of ‘Plant-pathogen interaction’ in the pink module likely reflects crosstalk between developmental and defense signaling pathways; many genes annotated under this pathway also participate in basal surveillance, cell wall remodeling, and redox homeostasis—processes recruited during normal development to support growth and senescence. The magenta module, strongly linked to yield, integrates MAPK signaling with ‘Amino sugar and nucleotide sugar metabolism’ and ‘Endocytosis’, bridging signal perception with metabolic execution and cellular restructuring for enhanced transport capacity.

Stage-specific transcription factor expression—suppression of defense-related WRKYs and MYBs at flowering and their strong induction during pod-filling—reflects developmental regulation of these transcriptional regulators. WRKYs, NACs, and ERFs/DREBs have established functions in senescence, secondary metabolism, and developmental reprogramming under non-stress conditions (Kim et al., 2018). ZAT10 upregulation in pod-filling tissues aligns with its roles in photosynthetic regulation and redox homeostasis during reproductive maturation. The induction of CBF/DREB transcription factors in this study occurs without low-temperature stress, consistent with their additional developmental functions in senescence and reproductive maturation (Wisniewski et al., 2014).

The temporal coordination of these TF programs and the systemic induction of the leaf nutrient reservoir program provides a regulatory framework for DA-6-mediated yield-quality synergy.

### 4.5 A unified model for yield and quality enhancement

Based on our integrative data, we propose a unified model for DA-6 action (**Figure 10**). DA-6 initiates its program during flowering, priming stem growth and metabolic infrastructure through auxin signaling and the yellow module. Concurrently, it initiates the ‘leaf priming’ phase, activating a seed-like program for temporary nutrient storage, as evidenced by the enrichment of aleurone grain-related genes and the expression of seed storage components in leaves.

During pod-filling, it activates two key, interconnected arms: firstly, in leaves (pink module), it enhances photosynthetic stability and stress resilience via MAPK signaling, ensuring a strong and durable source capacity, and now, critically, begins to remobilize the stored nutrients from its temporary reservoir; secondly, in stems (magenta module), it triggers a massive metabolic shift towards carbon mobilization and enhanced transport capacity. This entire process is underpinned by a temporally evolving hormonal dialogue.

By coordinately strengthening the source (leaf photosynthesis, resilience, and nutrient storage), optimizing the transport pathway (stem metabolism and vascular function), and strengthening the sink (via hormonal signals and balanced carbon and nitrogen delivery), DA-6 ensures a greater, more efficient, and more protected flow of photoassimilates and amino acids into seeds. This coordinated multi-organ effort results in higher yield without diluting the protein and oil content.

### 4.6 Limitations and future directions

While our study provides a comprehensive transcriptomic framework for DA-6-mediated synergy between yield and quality, several limitations should be acknowledged. First, our conclusions are primarily based on transcriptional evidence; validation at the protein, metabolite, and hormone levels is needed to fully establish causal relationships. For example, quantitative profiling of auxin, jasmonate, and cytokinin in different tissues across stages would strengthen the hormonal model. Second, although we validated key DEGs by qPCR, functional characterization of candidate hub genes (e.g., BAM3, TPS9, ABI3) through genetic manipulation is required to confirm their roles. Third, our study was conducted with a single soybean cultivar under non-stress conditions in one growing season. Future work should test the robustness of these mechanisms across diverse genotypes, environments, and stress scenarios. Finally, integrating metabolomics and proteomics with transcriptomics will provide a more holistic view of the metabolic fluxes and post-transcriptional regulation involved. Despite these limitations, our findings offer valuable candidate genes and modules for molecular breeding and highlight DA-6 as a promising tool for sustainable soybean intensification.

## 5 Conclusion

In conclusion, our study elucidates the complex transcriptional and regulatory networks that mediate DA-6’s synergistic effects. We demonstrate that DA-6 breaks the yield-quality trade-off by masterfully coordinating hormonal signals, metabolic pathways, and stress responses across time and space. It employs a “division of labor” among organs, priming them in developmental sequence to collectively support a highly efficient source-sink system. The discovery of the DA-6-induced ‘leaf priming’ mechanism, transforming leaves into temporary nutrient reservoirs, provides a novel and powerful explanation for the concurrent enhancement of yield and quality. This research provides a solid molecular theoretical foundation for employing DA-6 in high-yield, high-quality soybean cultivation and offers valuable candidate genes and modules for future genetic improvement.

## Data Availability

The datasets presented in this study can be found in online repositories. The names of the repository/repositories and accession number(s) can be found below: https://www.ncbi.nlm.nih.gov/sra/PRJNA1429446, accession number PRJNA1429446.
