# Supplementary material for: Synergistic enhancement of soybean yield and quality by diethyl aminoethyl hexanoate: unraveling the molecular mechanisms through integrated transcriptomics
Source: Front Plant Sci. 2026 Apr 10;17:1784831. doi: 10.3389/fpls.2026.1784831 (PMC13106087; doi:10.3389/fpls.2026.1784831)
Supplement: Supplementary file 1 [file Table1.docx]

**Table S1.** Summary of RNA-seq data

| Sample | Clean reads | Total mapped | Multiple mapped | Uniquely mapped |
| --- | --- | --- | --- | --- |
| T0ZD_L_1 | 50.13M | 48333014(96.42%) | 1623911(3.24%) | 46709103(93.18%) |
| T0ZD_L_2 | 50.66M | 49116494(96.95%) | 1619617(3.20%) | 47496877(93.75%) |
| T0ZD_L_3 | 46.59M | 45204216(97.02%) | 1503712(3.23%) | 43700504(93.79%) |
| T0ZD_P_1 | 48.60M | 47214144(97.14%) | 2849127(5.86%) | 44365017(91.28%) |
| T0ZD_P_2 | 48.58M | 46604261(95.94%) | 4092760(8.43%) | 42511501(87.51%) |
| T0ZD_P_3 | 47.65M | 46339017(97.25%) | 3485051(7.31%) | 42853966(89.93%) |
| T0ZD_S_1 | 46.78M | 44825121(95.82%) | 1086377(2.32%) | 43738744(93.50%) |
| T0ZD_S_2 | 48.26M | 46896757(97.18%) | 1123371(2.33%) | 45773386(94.85%) |
| T0ZD_S_3 | 45.85M | 44314503(96.66%) | 1098494(2.40%) | 43216009(94.26%) |
| T0ZD_R_L_1 | 45.20M | 40493072(89.59%) | 2618450(5.79%) | 37874622(83.80%) |
| T0ZD_R_L_2 | 43.04M | 41540277(96.52%) | 1081026(2.51%) | 40459251(94.01%) |
| T0ZD_R_L_3 | 42.88M | 41389250(96.53%) | 1084474(2.53%) | 40304776(94.00%) |
| T0ZD_R_P_1 | 43.26M | 42057761(97.21%) | 3346925(7.74% | 38710836(89.47%) |
| T0ZD_R_P_2 | 45.81M | 44327862(96.75%) | 4500165(9.82%) | 39827697(86.93%) |
| T0ZD_R_P_3 | 48.12M | 44786450(93.07%) | 4180336(8.69%) | 40606114(84.38%) |
| T0ZD_R_S_1 | 46.22M | 44945386(97.24%) | 1075732(2.32%) | 43896661(94.97%) |
| T0ZD_R_S_2 | 44.63M | 43339463(97.12%) | 1150729(2.28%) | 42352462(94.91%) |
| T0ZD_R_S_3 | 45.60M | 44404258(97.37%) | 1018966(2.32%) | 43335357(95.03%) |
| T1ZD_L_1 | 43.45M | 42099458(96.90%) | 1333242(3.07%) | 40766216(93.83%) |
| T1ZD_L_2 | 44.09M | 42783806(97.04%) | 1415773(3.21%) | 41368033(93.83%) |
| T1ZD_L_3 | 48.94M | 47264352(96.57%) | 1535905(3.14%) | 45728447(93.43%) |
| T1ZD_P_1 | 49.96M | 48513256(97.10%) | 3286040(6.58%) | 45227216(90.52%) |
| T1ZD_P_2 | 47.67M | 46226417(96.96%) | 4276460(8.97%) | 41949957(87.99%) |
| T1ZD_P_3 | 48.69M | 47208684(96.96%) | 3692150(7.58%) | 43516534(89.38%) |
| T1ZD_S_1 | 47.56M | 46021441(96.76%) | 1103458(2.32%) | 44917983(94.44%) |
| T1ZD_S_2 | 50.42M | 48699895(96.59%) | 1222390(2.42%) | 47477505(94.16%) |
| T1ZD_S_3 | 47.57M | 46165761(97.05%) | 1125748(2.37%) | 45040013(94.68%) |
| T1ZD_R_L_1 | 39.13M | 37648836(96.21%) | 1001906(2.56%) | 36646930(93.65%) |
| T1ZD_R_L_2 | 42.80M | 40986486(95.77%) | 1128675(2.64%) | 39857811(93.13%) |
| T1ZD_R_L_3 | 39.47M | 37861057(95.91%) | 1024720(2.60%) | 36836337(93.32%) |
| T1ZD_R_P_1 | 47.22M | 45530804(96.43%) | 4574531(9.69%) | 40956273(86.74%) |
| T1ZD_R_P_2 | 49.09M | 47515527(96.79%) | 4233338(8.62%) | 43282189(88.17%) |
| T1ZD_R_P_3 | 47.32M | 45556474(96.27%) | 4269855(9.02%) | 41286619(87.25%) |
| T1ZD_R_S_1 | 46.40M | 45174984(97.35%) | 1075732(2.32%) | 44099252(95.04%) |
| T1ZD_R_S_2 | 50.53M | 49055941(97.07%) | 1150729(2.28%) | 47905212(94.80%) |
| T1ZD_R_S_3 | 43.84M | 42557991(97.07%) | 1018966(2.32%) | 41539025(94.75%) |
