# Supplementary material for: Synergistic enhancement of soybean yield and quality by diethyl aminoethyl hexanoate: unraveling the molecular mechanisms through integrated transcriptomics
Source: Front Plant Sci. 2026 Apr 10;17:1784831. doi: 10.3389/fpls.2026.1784831 (PMC13106087; doi:10.3389/fpls.2026.1784831)
Supplement: Supplementary file 4 [file Table4.docx]

Table S4 DEGs involved in carbohydrate metabolism

T0ZD_L VS T1ZD_L:

| Glyma.20G112600 | Alpha-galactosidase 1；pectate lyase | up |
| --- | --- | --- |
| Glyma.20G040600 | Alcohol dehydrogenase-like 1 | up |
| Glyma.13G168700 | Aldehyde dehydrogenase family 3 member F1 | up |
| Glyma.16G014100 | Alcohol dehydrogenase 1 | up |
| Glyma.09G178100 | Formate dehydrogenase 1, mitochondrial | up |
| Glyma.14G156400 | alcohol dehydrogenase class-P (ADH1) | up |
| Glyma.06G120300 | Alcohol dehydrogenase 1 {ECO:0000303\|PubMed:3309331} | up |
| Glyma.14G211100 | Glutamate decarboxylase 1 | up |
| Glyma.19G124600 | Pectinesterase 3 | up |
| Glyma.12G015100 | Pectinesterase PPE8B | up |
| Glyma.06G314200 | Probable 2-isopropylmalate synthase | up |
| Glyma.09G273400 | Alpha-L-arabinofuranosidase 1 | up |
| Glyma.19G120400 | Non-specific phospholipase C2 | up |
| Glyma.13G035200 | Probable pectate lyase 12 | up |
| Glyma.20G164700 | Acidic endochitinase； chitinase | up |
| Glyma.03G229800 | Galactinol synthase 2 | down |
| Glyma.08G201200 | 2-oxoisovalerate dehydrogenase subunit alpha 2, mitochondrial | down |
| Glyma.12G057400 | Pectinesterase 47 | down |
| Glyma.14G077900 | NADP-dependent malic enzyme 4, chloroplastic | down |

T0ZD_P VS T1ZD_P：

| Glyma.13G346700 | Endochitinase PR4 | down |
| --- | --- | --- |
| Glyma.17G027300 | UDP-glucuronic acid decarboxylase 2 | down |
| Glyma.20G164900 | Acidic endochitinase | down |

T0ZD_S VS T1ZD_S：

| Glyma.01G160100 | Endochitinase | up |
| --- | --- | --- |
| Glyma.02G208000 | UDP-glucuronate 4-epimerase 1 | up |
| Glyma.02G292800 | GDP-L-galactose phosphorylase 1 | up |
| Glyma.03G055100 | Pyruvate decarboxylase 4 | up |
| Glyma.03G215800 | Pectinesterase 41 | up |
| Glyma.04G088000 | Inositol-tetrakisphosphate 1-kinase 3 | up |
| Glyma.05G136100 | Glutamate decarboxylase 4 | up |
| Glyma.06G089800 | Inositol-tetrakisphosphate 1-kinase 3 | up |
| Glyma.06G114300 | Aldehyde dehydrogenase family 3 member H1 | up |
| Glyma.07G266200 | Inositol monophosphatase 3 | up |
| Glyma.08G137500 | Probable trehalose-phosphate phosphatase J | up |
| Glyma.08G243000 | UDP-glucose 6-dehydrogenase 1 | up |
| Glyma.09G138100 | Oxalate--CoA ligase {ECO:0000305} | up |
| Glyma.09G168300 | Beta-amylase 1, chloroplastic | up |
| Glyma.10G145300 | Galactinol synthase 2 | up |
| Glyma.11G129500 | Beta-glucosidase 24 | up |
| Glyma.11G129700 | Beta-glucosidase 24 | up |
| Glyma.11G129800 | Beta-glucosidase 24 | up |
| Glyma.11G198300 | Oxalate--CoA ligase {ECO:0000305} | up |
| Glyma.13G088300 | Probable trehalose-phosphate phosphatase I | up |
| Glyma.14G171700 | Probable trehalose-phosphate phosphatase 4 | up |
| Glyma.16G014000 | Pectinesterase 3 | up |
| Glyma.17G069600 | UDP-glucuronate 4-epimerase 1 | up |
| Glyma.18G265100 | UDP-glucose 6-dehydrogenase 1 | up |
| Glyma.19G219100 |  | up |
| Glyma.20G051700 | L-ascorbate oxidase | up |
| Glyma.20G094500 | Galactinol synthase 2 | up |

T0ZD_R_L VS T1ZD_R_L：

| Glyma.11G129700 | Beta-glucosidase 24 | up |
| --- | --- | --- |
| Glyma.12G130200 | Beta-glucosidase 24 | up |
| Glyma.11G129600 | Beta-glucosidase 24 | up |
| Glyma.02G015800 | Fumarate hydratase 2, chloroplastic | up |
| Glyma.13G088300 | Probable trehalose-phosphate phosphatase I | up |
| Glyma.10G009000 | Pectinesterase 21 | up |
| Glyma.11G129500 | Beta-glucosidase 24 | up |
| Glyma.12G054100 | Beta-glucosidase 24 | up |
| Glyma.11G129800 | Beta-glucosidase 24 | up |
| Glyma.11G095600 | Hexokinase-4 | up |
| Glyma.11G116600 | Glucose-1-phosphate adenylyltransferase large subunit 1 | up |
| Glyma.03G215800 | Pectinesterase 41 | up |
| Glyma.20G051700 | L-ascorbate oxidase | up |
| Glyma.06G314200 | Pectinesterase 47 | up |
| Glyma.11G129900 | Beta-glucosidase 24 | up |
| Glyma.11G198300 | Oxalate--CoA ligase {ECO:0000305} | up |
| Glyma.08G138200 | Inositol-3-phosphate synthase | up |
| Glyma.18G272200 | UDP-arabinopyranose mutase 1 | up |
| Glyma.08G091300 | Glutamate decarboxylase 1 | up |
| Glyma.13G264500 | UDP-4-keto-6-deoxy-D-glucose 3,5-epimerase/UDP-4-keto-L-rhamnose 4-keto-reductase {ECO:0000305\|PubMed:17190829} | up |
| Glyma.09G138100 | Oxalate--CoA ligase {ECO:0000305} | up |
| Glyma.18G265100 | UDP-glucose 6-dehydrogenase 1 | up |
| Glyma.09G168300 | Beta-amylase 1, chloroplastic | up |
| Glyma.10G227700 | Acidic endochitinase | up |
| Glyma.06G087800 | NADP-dependent malic enzyme | up |
| Glyma.11G190200 | UDP-D-apiose/UDP-D-xylose synthase 2 | up |
| Glyma.13G076900 | L-ascorbate oxidase | up |
| Glyma.12G049100 | Endochitinase PR4 | up |
| Glyma.12G054200 | Beta-glucosidase 24 | up |
| Glyma.13G057700 | UDP-glucose 6-dehydrogenase 1 | up |
| Glyma.09G017000 | Probable galacturonosyltransferase 4 | up |
| Glyma.08G023100 | UDP-glucose 4-epimerase GEPI48 | up |
| Glyma.08G249900 | Alpha-1,4-glucan-protein synthase [UDP-forming] 1 | down |
| Glyma.01G005100 | Inositol oxygenase 2 | down |
| Glyma.06G310900 | 1,4-alpha-glucan-branching enzyme 1, chloroplastic/amyloplastic | down |
| Glyma.01G203400 | Beta-amylase 3, chloroplastic | down |
| Glyma.13G045700 | Mannose-6-phosphate isomerase 1 | down |
| Glyma.08G138100 | 2,3-bisphosphoglycerate-dependent phosphoglycerate mutase | down |
| Glyma.09G007900 | Pyrophosphate--fructose 6-phosphate 1-phosphotransferase subunit beta | down |
| Glyma.05G236800 | Pectinesterase 6 | down |
| Glyma.20G112600 | Probable pectate lyase 12 | down |
| Glyma.14G211100 | Glutamate decarboxylase 1 | down |
| Glyma.01G006800 | Probable pectinesterase 53 | down |

T0ZD_R_P VS T1ZD_R_P：

| Glyma.11G129500 | Beta-glucosidase 24 | up |
| --- | --- | --- |
| Glyma.01G118000 | Pyruvate decarboxylase 4 | up |
| Glyma.14G121200 | Alcohol dehydrogenase 1 {ECO:0000303\|PubMed:3309331} | up |
| Glyma.11G039400 | Beta-amylase 3, chloroplastic | up |
| Glyma.11G198300 | Oxalate--CoA ligase {ECO:0000305} | up |
| Glyma.05G036100 | Probable alpha,alpha-trehalose-phosphate synthase [UDP-forming] 9 | up |
| Glyma.05G224500 | Inositol oxygenase 4 | up |
| Glyma.08G180900 | Alpha-L-arabinofuranosidase | up |
| Glyma.13G053000 | UDP-arabinopyranose mutase 1 | up |
| Glyma.14G010900 | Fructose-bisphosphate aldolase, cytoplasmic isozyme 1 | down |
| Glyma.19G219100 | Galactinol synthase 1 | down |
| Glyma.11G111100 | Fructose-bisphosphate aldolase 1, chloroplastic | down |
| Glyma.13G060900 | Pectinesterase 31 | down |
| Glyma.14G161800 | Probable 6-phosphogluconolactonase 4, chloroplastic | down |

T0ZD_R_S VS T1ZD_R_S：

| Glyma.08G144400 | 2,3-bisphosphoglycerate-dependent phosphoglycerate mutase | up |
| --- | --- | --- |
| Glyma.08G137500 | Probable trehalose-phosphate phosphatase J | up |
| Glyma.04G243000 | 2-oxoisovalerate dehydrogenase subunit alpha 2, mitochondrial | up |
| Glyma.20G051700 | L-ascorbate oxidase | up |
| Glyma.08G205000 | Glyoxylate/hydroxypyruvate reductase A HPR2 | up |
| Glyma.18G265100 | UDP-glucose 6-dehydrogenase 1 | up |
| Glyma.06G318400 | Trehalose-phosphate phosphatase A | up |
| Glyma.11G095600 | Hexokinase-4 | up |
| Glyma.05G179800 | Probable trehalose-phosphate phosphatase J | up |
| Glyma.08G243000 | UDP-glucose 6-dehydrogenase 1 | up |
| Glyma.11G198300 | Oxalate--CoA ligase {ECO:0000305} | up |
| Glyma.11G129500 | Beta-glucosidase 24 | up |
| Glyma.16G014000 | Pectinesterase 3 | up |
| Glyma.13G076900 | L-ascorbate oxidase | up |
| Glyma.11G129700 | Beta-glucosidase 24 | up |
| Glyma.12G054100 | Beta-glucosidase 24 | up |
| Glyma.18G224200 | Polygalacturonate 4-alpha-galacturonosyltransferase | up |
| Glyma.01G215500 | Hydroxymethylglutaryl-CoA synthase | up |
| Glyma.11G190200 | UDP-D-apiose/UDP-D-xylose synthase 2 | up |
| Glyma.12G130200 | Beta-glucosidase 24 | up |
| Glyma.06G089800 | Inositol-tetrakisphosphate 1-kinase 3 | up |
| Glyma.07G266200 | Inositol monophosphatase 3 | up |
| Glyma.14G214300 | Glucan endo-1,3-beta-glucosidase 2 | up |
| Glyma.20G051900 | L-ascorbate oxidase | up |
| Glyma.11G129800 | Beta-glucosidase 24 | up |
| Glyma.06G087800 | NADP-dependent malic enzyme | up |
| Glyma.11G129600 | Beta-glucosidase 24 | up |
| Glyma.12G087900 | UDP-D-apiose/UDP-D-xylose synthase 2 | up |
| Glyma.09G232900 | UDP-glucuronate 4-epimerase 4 | up |
| Glyma.20G164600 | Lysozyme | up |
| Glyma.09G138100 | Oxalate--CoA ligase {ECO:0000305} | up |
| Glyma.01G203400 | Beta-amylase 3, chloroplastic | up |
| Glyma.05G058100 | L-lactate dehydrogenase A | up |
| Glyma.13G264500 | UDP-4-keto-6-deoxy-D-glucose 3,5-epimerase/UDP-4-keto-L-rhamnose 4-keto-reductase | up |
| Glyma.06G184200 | Probable alpha,alpha-trehalose-phosphate synthase [UDP-forming] 9 | up |
| Glyma.20G125200 | Probable ribose-5-phosphate isomerase 2 | up |
| Glyma.15G122600 | Probable galacturonosyltransferase 4 | up |
| Glyma.01G063000 | Alpha-1,4-glucan-protein synthase [UDP-forming] | up |
| Glyma.19G020700 | Aldose 1-epimerase | up |
| Glyma.09G266300 | Probable fructokinase-7 | up |
| Glyma.13G134200 | Probable pectate lyase 4 | up |
| Glyma.10G265400 | Probable ribose-5-phosphate isomerase 2 | up |
| Glyma.11G075700 | GDP-mannose 4,6 dehydratase 1 | up |
| Glyma.17G069600 | UDP-glucuronate 4-epimerase 1 | up |
| Glyma.09G233000 | Probable polygalacturonase | up |
| Glyma.12G180300 | UDP-D-apiose/UDP-D-xylose synthase 2 | up |
| Glyma.08G023100 | UDP-glucose 4-epimerase GEPI48 | up |
| Glyma.13G088300 | Probable trehalose-phosphate phosphatase I | up |
| Glyma.06G114300 | Aldehyde dehydrogenase family 3 member H1 | up |
| Glyma.09G077100 | Pectinesterase PPE8B | up |
| Glyma.19G158700 | Type IV inositol polyphosphate 5-phosphatase 7 {ECO:0000303\|PubMed:21677096} | up |
| Glyma.05G156000 | Glucan endo-1,3-beta-glucosidase 4 | up |
| Glyma.04G074000 | Glucan endo-1,3-beta-glucosidase 5 | up |
| Glyma.16G200600 | Probable fructokinase-7 | up |
| Glyma.01G167500 | GDP-mannose 4,6 dehydratase 2 | up |
| Glyma.13G278600 | Beta-glucosidase 18 | up |
| Glyma.18G204200 | Pyruvate decarboxylase 1 | up |
| Glyma.01G225800 | UDP-glucose 4-epimerase GEPI48 | up |
| Glyma.05G204700 | UDP-glucose 4-epimerase | up |
| Glyma.16G118000 | Polygalacturonase | up |
| Glyma.05G056300 | Acid beta-fructofuranosidase 70 kDa monomer | down |
| Glyma.12G156600 | Acidic endochitinase | down |
