# Supplementary material for: Synergistic enhancement of soybean yield and quality by diethyl aminoethyl hexanoate: unraveling the molecular mechanisms through integrated transcriptomics
Source: Front Plant Sci. 2026 Apr 10;17:1784831. doi: 10.3389/fpls.2026.1784831 (PMC13106087; doi:10.3389/fpls.2026.1784831)
Supplement: Supplementary file 5 [file Table5.docx]

Table S5 Differentially expressed transcription factors

T0ZD_L VS T1ZD_L:

| gene_id | Regulation | Description | Family |
| --- | --- | --- | --- |
| Glyma.08G357600 | up | B3 domain-containing transcription factor ABI3 | B3 |
| Glyma.18G176100 | up | B3 domain-containing transcription factor ABI3 | B3 |
| Glyma.01G019700 | up | Transcription factor bHLH93 | bHLH |
| Glyma.01G076900 | down | Transcription factor UNE10 | bHLH |
| Glyma.05G110900 | up | Transcription factor bHLH25 | bHLH |
| Glyma.07G117500 | up | Transcription factor bHLH25 | bHLH |
| Glyma.09G203000 | up | Transcription factor bHLH93 | bHLH |
| Glyma.04G029600 | up | bZIP transcription factor 44 | bZIP |
| Glyma.15G085400 | up | Ethylene-responsive transcription factor RAP2-11 | ERF |
| Glyma.17G143900 | up | Ethylene-responsive transcription factor ERF034 | ERF |
| Glyma.17G210500 | down | Ethylene-responsive transcription factor SHINE 2 | ERF |
| Glyma.11G136600 | up | Transcription factor LUX | G2-like |
| Glyma.12G060200 | up | Transcription factor LUX | G2-like |
| Glyma.15G083600 | down | Putative Myb family transcription factor | G2-like |
| Glyma.19G110200 | up | GATA transcription factor 9 | GATA |
| Glyma.10G237800 | down | Heat stress transcription factor B-2a | HSF |
| Glyma.17G053700 | up | Heat stress transcription factor A-2 | HSF |
| Glyma.10G142200 | up | Transcription factor MYB86 | MYB |
| Glyma.13G247200 | up | Transcription factor MYB39 | MYB |
| Glyma.03G191700 | up | Trihelix transcription factor PTL | Trihelix |
| Glyma.13G313000 | up | Transcription factor LHW | bHLH |

T0ZD_P VS T1ZD_P：

| gene_id | Regulation | Description | Family |
| --- | --- | --- | --- |
| Glyma.04G020000 | Down | High mobility group B protein 9 | ARID-HMG1 |
| Glyma.11G018100 | up | Ethylene-responsive transcription factor ERF061 | ERF |
| Glyma.20G155200 | Down | Ethylene-responsive transcription factor ERF025 | ERF |

T0ZD_S VS T1ZD_S：

| gene_id | Regulation | Description | Family |
| --- | --- | --- | --- |
| Glyma.13G266600 | down | BES1/BZR1 homolog protein 4 | BES1 |
| Glyma.01G197900 | down | Transcription factor bHLH25 | bHLH |
| Glyma.02G000800 | down | Transcription factor bHLH92 | bHLH |
| Glyma.04G090100 | up | Transcription factor bHLH93 | bHLH |
| Glyma.05G110600 | down | Transcription factor bHLH25 | bHLH |
| Glyma.08G271900 | down | Transcription factor MYC2 | bHLH |
| Glyma.09G060200 | down | Transcription factor bHLH35 | bHLH |
| Glyma.09G204500 | down | Transcription factor MYC2 | bHLH |
| Glyma.11G043700 | down | Transcription factor bHLH25 | bHLH |
| Glyma.12G114300 | down | Transcription factor bHLH36 | bHLH |
| Glyma.13G101100 | down | Transcription factor bHLH35 | bHLH |
| Glyma.15G064500 | down | Transcription factor bHLH123 | bHLH |
| Glyma.15G166800 | down | Transcription factor bHLH35 | bHLH |
| Glyma.17G058600 | down | Transcription factor bHLH35 | bHLH |
| Glyma.02G161100 | down | bZIP transcription factor 50 | bZIP |
| Glyma.10G013300 | down | bZIP transcription factor 53 | bZIP |
| Glyma.14G217200 | down | Transcription factor VIP1 | bZIP |
| Glyma.17G255800 | down | Transcription factor VIP1 | bZIP |
| Glyma.04G044900 | down | Zinc finger protein ZAT10 | C2H2 |
| Glyma.06G287200 | up | Zinc finger protein NUTCRACKER | C2H2 |
| Glyma.10G045300 | down | Zinc finger protein ZAT11 | C2H2 |
| Glyma.10G045400 | down | Zinc finger protein ZAT11 | C2H2 |
| Glyma.10G257900 | down | Zinc finger protein AZF1 | C2H2 |
| Glyma.11G142500 | down | Zinc finger protein ZAT8 | C2H2 |
| Glyma.13G133000 | down | Zinc finger protein ZAT11 | C2H2 |
| Glyma.13G133100 | down | Zinc finger protein ZAT11 | C2H2 |
| Glyma.13G333400 | down | Zinc finger protein ZAT11 | C2H2 |
| Glyma.14G088300 | down | Zinc finger protein ZAT10 | C2H2 |
| Glyma.14G110900 | up | Zinc finger protein 3 | C2H2 |
| Glyma.15G040700 | down | Zinc finger protein ZAT12 | C2H2 |
| Glyma.17G064700 | up | Zinc finger protein 2 | C2H2 |
| Glyma.17G236200 | down | Zinc finger protein ZAT10 | C2H2 |
| Glyma.19G174200 | down | Zinc finger protein ZAT11 | C2H2 |
| Glyma.02G296600 | down | Zinc finger CCCH domain-containing protein 29 | C3H |
| Glyma.03G138600 | down | Zinc finger CCCH domain-containing protein 30 | C3H |
| Glyma.14G016300 | down | Zinc finger CCCH domain-containing protein 29 | C3H |
| Glyma.08G135200 | down | Calmodulin-binding transcription activator 2 | CAMTA |
| Glyma.13G241900 | down | Dof zinc finger protein DOF1.7 | Dof |
| Glyma.01G206600 | down | Ethylene-responsive transcription factor 1A | ERF |
| Glyma.01G206700 | down | Ethylene-responsive transcription factor 6 | ERF |
| Glyma.01G216000 | down | Dehydration-responsive element-binding protein 1F | ERF |
| Glyma.01G224100 | down | Ethylene-responsive transcription factor CRF4 | ERF |
| Glyma.01G231000 | down | Ethylene-responsive transcription factor ERF022 | ERF |
| Glyma.01G231200 | down | Ethylene-responsive transcription factor ERF021 | ERF |
| Glyma.02G016100 | down | Ethylene-responsive transcription factor RAP2-3 | ERF |
| Glyma.02G080200 | down | Ethylene-responsive transcription factor ERF110 | ERF |
| Glyma.02G132500 | down | Ethylene-responsive transcription factor ERF112 | ERF |
| Glyma.02G261700 | down | Dehydration-responsive element-binding protein 2C | ERF |
| Glyma.02G294100 | down | Ethylene-responsive transcription factor 7 | ERF |
| Glyma.03G159800 | down | Ethylene-responsive transcription factor 3 | ERF |
| Glyma.03G191800 | down | Ethylene-responsive transcription factor ERF024 | ERF |
| Glyma.04G057700 | down | Ethylene-responsive transcription factor ERF018 | ERF |
| Glyma.04G251400 | down | Ethylene-responsive transcription factor ERF012 | ERF |
| Glyma.05G049800 | down | Ethylene-responsive transcription factor ERF026 | ERF |
| Glyma.05G049900 | down | Dehydration-responsive element-binding protein 1E | ERF |
| Glyma.06G058400 | down | Ethylene-responsive transcription factor ERF018 | ERF |
| Glyma.06G111300 | down | Ethylene-responsive transcription factor ERF012 | ERF |
| Glyma.06G125100 | down | Ethylene-responsive transcription factor CRF4 | ERF |
| Glyma.06G236400 | down | Ethylene-responsive transcription factor 4 | ERF |
| Glyma.06G290000 | down | Ethylene-responsive transcription factor 9 | ERF |
| Glyma.07G113800 | down | Ethylene-responsive transcription factor 2 | ERF |
| Glyma.07G212400 | down | Ethylene-responsive transcription factor ERF109 | ERF |
| Glyma.08G035000 | down | Dehydration-responsive element-binding protein 3 | ERF |
| Glyma.09G072000 | down | Ethylene-responsive transcription factor ERF017 | ERF |
| Glyma.09G147200 | down | Dehydration-responsive element-binding protein 1D | ERF |
| Glyma.09G242600 | down | Ethylene-responsive transcription factor 1 | ERF |
| Glyma.10G016500 | down | Ethylene-responsive transcription factor RAP2-3 | ERF |
| Glyma.10G066900 | down | Ethylene-responsive transcription factor ERF024 | ERF |
| Glyma.11G014200 | down | Ethylene-responsive transcription factor ERF021 | ERF |
| Glyma.11G036400 | down | Ethylene-responsive transcription factor 5 | ERF |
| Glyma.11G036500 | down | Ethylene-responsive transcription factor 1A | ERF |
| Glyma.11G199300 | down | Pathogenesis-related genes transcriptional activator PTI6 | ERF |
| Glyma.12G117000 | down | Ethylene-responsive transcription factor 9 | ERF |
| Glyma.12G162700 | down | Ethylene-responsive transcription factor 9 | ERF |
| Glyma.12G226600 | down | Ethylene-responsive transcription factor 4 | ERF |
| Glyma.13G088100 | down | Ethylene-responsive transcription factor RAP2-4 | ERF |
| Glyma.13G112400 | down | Ethylene-responsive transcription factor ERF017 | ERF |
| Glyma.13G151900 | down | Ethylene-responsive transcription factor ERF024 | ERF |
| Glyma.13G236500 | down | Ethylene-responsive transcription factor 4 | ERF |
| Glyma.13G236600 | down | Ethylene-responsive transcription factor 12 | ERF |
| Glyma.13G274100 | down | Ethylene-responsive transcription factor 9 | ERF |
| Glyma.13G298600 | down | Ethylene-responsive transcription factor ERF053 | ERF |
| Glyma.14G020100 | down | Ethylene-responsive transcription factor 3 | ERF |
| Glyma.14G056200 | down | Dehydration-responsive element-binding protein 2C | ERF |
| Glyma.14G106200 | down | Ethylene-responsive transcription factor ERF017 | ERF |
| Glyma.14G147500 | down | Ethylene-responsive transcription factor ERF012 | ERF |
| Glyma.14G171500 | down | Ethylene-responsive transcription factor RAP2-4 | ERF |
| Glyma.15G025100 | down | Ethylene-responsive transcription factor ERF024 | ERF |
| Glyma.15G077100 | down | Ethylene-responsive transcription factor 4 | ERF |
| Glyma.15G180000 | down | Ethylene-responsive transcription factor ERF017 | ERF |
| Glyma.16G199000 | down | Dehydration-responsive element-binding protein 1D | ERF |
| Glyma.17G047300 | down | Ethylene-responsive transcription factor ERF017 | ERF |
| Glyma.17G131800 | down | Ethylene-responsive transcription factor ERF025 | ERF |
| Glyma.17G131900 | down | Dehydration-responsive element-binding protein 1C | ERF |
| Glyma.17G145400 | down | Ethylene-responsive transcription factor 1A | ERF |
| Glyma.18G252300 | down | Ethylene-responsive transcription factor 13 | ERF |
| Glyma.19G192400 | down | Ethylene-responsive transcription factor ERF024 | ERF |
| Glyma.20G070000 | down | Ethylene-responsive transcription factor ERF105 | ERF |
| Glyma.20G115300 | down | Ethylene-responsive transcription factor ERF118 | ERF |
| Glyma.20G196400 | down | Ethylene-responsive transcription factor 1 | ERF |
| Glyma.04G008900 | down | GATA transcription factor 7 | GATA |
| Glyma.07G108900 | down | GATA transcription factor 2 | GATA |
| Glyma.10G210500 | down | GATA transcription factor 9 | GATA |
| Glyma.12G037700 | down | GATA transcription factor 7 | GATA |
| Glyma.06G265500 | down | Chitin-inducible gibberellin-responsive protein 1 | GRAS |
| Glyma.07G266500 | down | Scarecrow-like protein 13 | GRAS |
| Glyma.09G011800 | down | Scarecrow-like protein 13 | GRAS |
| Glyma.09G133600 | down | Scarecrow-like protein 8 | GRAS |
| Glyma.11G138000 | down | Scarecrow-like protein 14 | GRAS |
| Glyma.12G061900 | down | Scarecrow-like protein 14 | GRAS |
| Glyma.12G137700 | down | Chitin-inducible gibberellin-responsive protein 1 | GRAS |
| Glyma.12G216100 | down | Chitin-inducible gibberellin-responsive protein 1 | GRAS |
| Glyma.13G285400 | down | Chitin-inducible gibberellin-responsive protein 1 | GRAS |
| Glyma.15G116300 | down | Scarecrow-like protein 13 | GRAS |
| Glyma.17G007600 | down | Scarecrow-like protein 13 | GRAS |
| Glyma.18G081100 | down | Scarecrow-like protein 5 | GRAS |
| Glyma.05G062700 | up | Homeobox-leucine zipper protein HAT4 | HD-ZIP |
| Glyma.07G052100 | down | Homeobox-leucine zipper protein ATHB-12 | HD-ZIP |
| Glyma.09G239400 | up | Homeobox-leucine zipper protein HAT7 | HD-ZIP |
| Glyma.13G102800 | down | Homeobox-leucine zipper protein HAT3 | HD-ZIP |
| Glyma.15G168800 | down | Homeobox-leucine zipper protein HAT22 | HD-ZIP |
| Glyma.17G144700 | up | Homeobox-leucine zipper protein HAT4 | HD-ZIP |
| Glyma.01G185800 | down | Heat shock factor protein HSF24 | HSF |
| Glyma.01G217400 | down | Heat stress transcription factor B-2b | HSF |
| Glyma.04G052000 | down | Heat shock factor protein HSF30 | HSF |
| Glyma.09G143200 | down | Heat stress transcription factor B-2a | HSF |
| Glyma.10G003100 | down | Heat stress transcription factor A-6b | HSF |
| Glyma.10G029600 | down | Heat stress transcription factor A-6b | HSF |
| Glyma.10G066100 | down | Heat stress transcription factor A-3 | HSF |
| Glyma.10G237800 | down | Heat stress transcription factor B-2a | HSF |
| Glyma.11G056200 | down | Heat shock factor protein HSF24 | HSF |
| Glyma.13G151200 | down | Heat stress transcription factor A-3 | HSF |
| Glyma.14G096800 | down | Heat shock factor protein HSF30 | HSF |
| Glyma.20G156800 | down | Heat stress transcription factor B-2a | HSF |
| Glyma.01G016600 | down | Transcription factor MYB39 | MYB |
| Glyma.02G005600 | down | Transcription factor MYB14 | MYB |
| Glyma.03G221700 | down | Transcription factor MYB108 | MYB |
| Glyma.04G036700 | down | Transcription factor MYB44 | MYB |
| Glyma.04G042300 | down | Transcription factor MYB44 | MYB |
| Glyma.05G098200 | down | Transcription factor MYB44 | MYB |
| Glyma.06G036800 | down | Transcription factor MYB44 | MYB |
| Glyma.06G300200 | down | Transcription factor MYB14 | MYB |
| Glyma.07G228700 | up | Transcription factor MYB56 | MYB |
| Glyma.19G219000 | up | Transcription factor MYB62 | MYB |
| Glyma.20G090700 | up | Transcription factor MYB86 | MYB |
| Glyma.20G209700 | down | Transcription factor MYB14 | MYB |
| Glyma.09G167900 | down | Protein REVEILLE 7 | MYB_related |
| Glyma.12G104800 | down | Transcription factor MYB14 | MYB_related |
| Glyma.18G044200 | down | Protein REVEILLE 1 | MYB_related |
| Glyma.20G111800 | down | Transcription factor SRM1 | MYB_related |
| Glyma.03G197900 | down | NAC domain-containing protein 90 | NAC |
| Glyma.04G208300 | down | NAC domain-containing protein 2 | NAC |
| Glyma.04G249000 | down | NAC domain-containing protein 2 | NAC |
| Glyma.05G195000 | down | NAC domain-containing protein 2 | NAC |
| Glyma.06G114000 | down | NAC domain-containing protein 2 | NAC |
| Glyma.06G157400 | down | NAC domain-containing protein 2 | NAC |
| Glyma.11G096600 | down | NAC domain-containing protein 35 | NAC |
| Glyma.11G182000 | down | NAC domain-containing protein 90 | NAC |
| Glyma.12G022700 | down | NAC domain-containing protein 35 | NAC |
| Glyma.12G091200 | down | NAC domain-containing protein 90 | NAC |
| Glyma.12G186200 | down | NAC domain-containing protein 90 | NAC |
| Glyma.13G315300 | down | NAC domain-containing protein 90 | NAC |
| Glyma.14G152700 | down | NAC domain-containing protein 2 | NAC |
| Glyma.16G016400 | down | NAC domain-containing protein 62 | NAC |
| Glyma.16G042900 | down | NAC domain-containing protein 100 | NAC |
| Glyma.16G069300 | down | SUPPRESSOR OF GAMMA RESPONSE 1 | NAC |
| Glyma.16G152100 | down | NAC domain-containing protein 79 | NAC |
| Glyma.17G185000 | down | NAC domain-containing protein 83 | NAC |
| Glyma.19G195800 | down | NAC domain-containing protein 90 | NAC |
| Glyma.20G172100 | down | NAC domain-containing protein 17 | NAC |
| Glyma.08G165700 | down | Nuclear transcription factor Y subunit C-2 | NF-YC |
| Glyma.15G261300 | down | Nuclear transcription factor Y subunit C-2 | NF-YC |
| Glyma.01G087500 | down | AP2/ERF and B3 domain-containing transcription repressor TEM1 | RAV |
| Glyma.02G099500 | down | AP2/ERF and B3 domain-containing transcription repressor RAV2 | RAV |
| Glyma.10G204400 | down | AP2/ERF and B3 domain-containing transcription repressor TEM1 | RAV |
| Glyma.05G237200 | down | Protein SAR DEFICIENT 1 | SARD1 |
| Glyma.04G134200 | down | Sigma factor binding protein 1, chloroplastic | SIB1 |
| Glyma.20G154400 | down | Transcription factor TCP19 | TCP |
| Glyma.01G121000 | down | Trihelix transcription factor GT-3a | Trihelix |
| Glyma.01G128100 | down | Probable WRKY transcription factor 33 | WRKY |
| Glyma.01G224800 | down | Probable WRKY transcription factor 41 | WRKY |
| Glyma.02G232600 | down | WRKY transcription factor WRKY24 | WRKY |
| Glyma.03G042700 | down | Probable WRKY transcription factor 33 | WRKY |
| Glyma.03G256700 | down | Probable WRKY transcription factor 41 | WRKY |
| Glyma.04G054200 | down | Probable WRKY transcription factor 50 | WRKY |
| Glyma.04G218700 | down | Probable WRKY transcription factor 51 | WRKY |
| Glyma.04G223300 | down | Probable WRKY transcription factor 70 | WRKY |
| Glyma.04G238300 | down | Probable WRKY transcription factor 30 | WRKY |
| Glyma.05G096500 | down | Probable WRKY transcription factor 15 | WRKY |
| Glyma.05G184500 | down | Probable WRKY transcription factor 51 | WRKY |
| Glyma.05G215900 | down | Probable WRKY transcription factor 41 | WRKY |
| Glyma.06G061900 | down | Probable WRKY transcription factor 40 | WRKY |
| Glyma.06G125600 | down | Probable WRKY transcription factor 30 | WRKY |
| Glyma.06G142000 | down | Probable WRKY transcription factor 70 | WRKY |
| Glyma.06G147100 | down | Probable WRKY transcription factor 51 | WRKY |
| Glyma.07G023300 | down | Probable WRKY transcription factor 40 | WRKY |
| Glyma.07G057400 | down | Probable WRKY transcription factor 41 | WRKY |
| Glyma.07G262700 | down | WRKY transcription factor 6 | WRKY |
| Glyma.08G021900 | down | Probable WRKY transcription factor 41 | WRKY |
| Glyma.08G142400 | down | Probable WRKY transcription factor 51 | WRKY |
| Glyma.08G218600 | down | Probable WRKY transcription factor 40 | WRKY |
| Glyma.09G061900 | down | Probable WRKY transcription factor 11 | WRKY |
| Glyma.09G274000 | down | Probable WRKY transcription factor 70 | WRKY |
| Glyma.09G280200 | down | Probable WRKY transcription factor 33 | WRKY |
| Glyma.11G163300 | down | WRKY transcription factor WRKY24 | WRKY |
| Glyma.13G370100 | down | Probable WRKY transcription factor 40 | WRKY |
| Glyma.14G103100 | down | Probable WRKY transcription factor 40 | WRKY |
| Glyma.14G200200 | down | WRKY transcription factor WRKY24 | WRKY |
| Glyma.15G003300 | down | Probable WRKY transcription factor 40 | WRKY |
| Glyma.15G110300 | down | WRKY transcription factor 6 | WRKY |
| Glyma.16G026400 | down | Probable WRKY transcription factor 41 | WRKY |
| Glyma.17G168900 | down | Probable WRKY transcription factor 15 | WRKY |
| Glyma.17G222300 | down | Probable WRKY transcription factor 40 | WRKY |
| Glyma.17G224800 | down | Probable WRKY transcription factor 50 | WRKY |
| Glyma.18G208800 | down | Probable WRKY transcription factor 33 | WRKY |
| Glyma.18G213200 | down | Probable WRKY transcription factor 70 | WRKY |
| Glyma.19G254800 | down | Probable WRKY transcription factor 41 | WRKY |

T0ZD_R_L VS T1ZD_R_L：

| gene_id | Regulation | Description | Family |
| --- | --- | --- | --- |
| Glyma.01G121000 | up | Trihelix transcription factor GT-3a | Trihelix |
| Glyma.01G128100 | up | Probable WRKY transcription factor 33 | WRKY |
| Glyma.01G188600 | down | Ethylene-responsive transcription factor ERF010 | ERF |
| Glyma.01G197900 | up | Transcription factor bHLH25 | bHLH |
| Glyma.01G216000 | up | Dehydration-responsive element-binding protein 1F | ERF |
| Glyma.01G225000 | up | Ethylene-responsive transcription factor ERF061 | ERF |
| Glyma.01G231000 | up | Ethylene-responsive transcription factor ERF022 | ERF |
| Glyma.01G231200 | up | Ethylene-responsive transcription factor ERF021 | ERF |
| Glyma.02G000800 | up | Transcription factor bHLH92 | bHLH |
| Glyma.02G016100 | up | Ethylene-responsive transcription factor RAP2-3 | ERF |
| Glyma.02G132500 | up | Ethylene-responsive transcription factor ERF112 | ERF |
| Glyma.02G210500 | up | Transcription factor bHLH96 | bHLH |
| Glyma.02G223700 | down | Zinc finger protein CONSTANS-LIKE 10 | CO-like |
| Glyma.02G296600 | up | Zinc finger CCCH domain-containing protein 29 | C3H |
| Glyma.02G306200 | up | Scarecrow-like transcription factor PAT1 | GRAS |
| Glyma.03G042700 | up | Probable WRKY transcription factor 33 | WRKY |
| Glyma.03G224700 | up | WRKY transcription factor 42 | WRKY |
| Glyma.03G256700 | up | Probable WRKY transcription factor 41 | WRKY |
| Glyma.04G008900 | up | GATA transcription factor 7 | GATA |
| Glyma.04G036700 | up | Transcription factor MYB44 | MYB |
| Glyma.04G057700 | up | Ethylene-responsive transcription factor ERF018 | ERF |
| Glyma.04G134200 | up | Sigma factor binding protein 1, chloroplastic | SIB1 |
| Glyma.04G208300 | up | NAC domain-containing protein 2 | NAC |
| Glyma.04G218400 | down | Probable WRKY transcription factor 13 | WRKY |
| Glyma.04G238300 | up | Probable WRKY transcription factor 30 | WRKY |
| Glyma.05G049800 | up | Ethylene-responsive transcription factor ERF026 | ERF |
| Glyma.05G049900 | up | Dehydration-responsive element-binding protein 1E | ERF |
| Glyma.05G110600 | up | Transcription factor bHLH25 | bHLH |
| Glyma.05G195000 | up | NAC domain-containing protein 2 | NAC |
| Glyma.05G215900 | up | Probable WRKY transcription factor 41 | WRKY |
| Glyma.05G237200 | up | Protein SAR DEFICIENT 1 | SARD |
| Glyma.06G009100 | down | B-box zinc finger protein 21 | DBB |
| Glyma.06G036800 | up | Transcription factor MYB44 | MYB |
| Glyma.06G045400 | up | Zinc finger protein ZAT10 | C2H2 |
| Glyma.06G058400 | up | Ethylene-responsive transcription factor ERF018 | ERF |
| Glyma.06G061900 | up | Probable WRKY transcription factor 40 | WRKY |
| Glyma.06G124300 | up | Dof zinc finger protein DOF3.5 | Dof |
| Glyma.06G125600 | up | Probable WRKY transcription factor 30 | WRKY |
| Glyma.06G142000 | up | Probable WRKY transcription factor 70 | WRKY |
| Glyma.06G157400 | up | NAC domain-containing protein 2 | NAC |
| Glyma.06G178600 | up | Transcription factor MYB86 | MYB |
| Glyma.06G255300 | up | B-box zinc finger protein 22 | BBX22 |
| Glyma.06G265500 | up | Chitin-inducible gibberellin-responsive protein 1 | GRAS |
| Glyma.06G290000 | up | Ethylene-responsive transcription factor 9 | ERF |
| Glyma.06G291900 | up | Transcription factor bHLH36 | bHLH |
| Glyma.06G299900 | up | Transcription factor MYB14 | MYB |
| Glyma.06G300100 | up | Transcription factor MYB14 | MYB |
| Glyma.06G300200 | up | Transcription factor MYB14 | MYB |
| Glyma.07G023300 | up | Probable WRKY transcription factor 40 | WRKY |
| Glyma.07G044800 | up | Agamous-like MADS-box protein AGL62 | AGL62 |
| Glyma.07G057400 | up | Probable WRKY transcription factor 41 | WRKY |
| Glyma.07G108900 | up | GATA transcription factor 2 | GATA |
| Glyma.07G113800 | up | Ethylene-responsive transcription factor 2 | ERF |
| Glyma.07G262700 | up | WRKY transcription factor 6 | WRKY |
| Glyma.08G021900 | up | Probable WRKY transcription factor 41 | WRKY |
| Glyma.08G035000 | up | Dehydration-responsive element-binding protein 3 | ERF |
| Glyma.08G044400 | up | Protein SAR DEFICIENT 1 | SARD1 |
| Glyma.08G056800 | down | Protein indeterminate-domain 14 | C2H2 |
| Glyma.08G135200 | up | Calmodulin-binding transcription activator 2 | CAMTA |
| Glyma.08G141000 | up | Nuclear transcription factor Y subunit B-3 | NF-YB |
| Glyma.08G152500 | up | Transcription factor bHLH130 | bHLH |
| Glyma.08G218600 | up | Probable WRKY transcription factor 40 | WRKY |
| Glyma.08G286500 | down | Transcription factor bHLH90 | bHLH |
| Glyma.09G147200 | up | Dehydration-responsive element-binding protein 1D | ERF |
| Glyma.09G241800 | up | Homeobox-leucine zipper protein HAT4 | HD-ZIP |
| Glyma.09G280200 | up | Probable WRKY transcription factor 33 | WRKY |
| Glyma.10G010400 | up | Transcription factor MYB78 | MYB |
| Glyma.10G016500 | up | Ethylene-responsive transcription factor RAP2-3 | ERF |
| Glyma.10G045300 | up | Zinc finger protein ZAT11 | C2H2 |
| Glyma.10G066100 | up | Heat stress transcription factor A-3 | HSF |
| Glyma.10G161200 | down | Trihelix transcription factor GTL2 | GTL2 |
| Glyma.10G180800 | up | Transcription factor MYB14 | MYB |
| Glyma.10G210500 | up | GATA transcription factor 9 | GATA |
| Glyma.10G237800 | up | Heat stress transcription factor B-2a | HSF |
| Glyma.10G239400 | up | Dehydration-responsive element-binding protein 1D | ERF |
| Glyma.11G009400 | down | Transcription factor MYB39 | MYB |
| Glyma.11G014200 | up | Ethylene-responsive transcription factor ERF021 | ERF |
| Glyma.11G036500 | up | Ethylene-responsive transcription factor 1A | ERF |
| Glyma.11G043700 | up | Transcription factor bHLH25 | bHLH |
| Glyma.11G096600 | up | NAC domain-containing protein 35 | NAC |
| Glyma.11G111700 | up | GATA transcription factor 7 | GATA |
| Glyma.11G112800 | down | B-box zinc finger protein 21 | DBB |
| Glyma.11G131800 | down | Serine/threonine-protein kinase UCN | UNC |
| Glyma.11G182000 | up | NAC domain-containing protein 90 | NAC |
| Glyma.12G022700 | up | NAC domain-containing protein 35 | NAC |
| Glyma.12G037700 | up | GATA transcription factor 7 | GATA |
| Glyma.12G061900 | up | Scarecrow-like protein 14 | GRAS |
| Glyma.12G091200 | up | NAC domain-containing protein 90 | NAC |
| Glyma.12G104600 | up | Transcription factor MYB14 | MYB |
| Glyma.12G104800 | up | Transcription factor MYB14 | MYB_related |
| Glyma.12G105400 | up | Transcription factor MYB14 | MYB |
| Glyma.12G114300 | up | Transcription factor bHLH36 | bHLH |
| Glyma.12G117000 | up | Ethylene-responsive transcription factor 9 | ERF |
| Glyma.12G186200 | up | NAC domain-containing protein 90 | NAC |
| Glyma.13G101100 | up | Transcription factor bHLH35 | bHLH |
| Glyma.13G102000 | up | Probable WRKY transcription factor 11 | WRKY |
| Glyma.13G117600 | up | WRKY transcription factor 42 | WRKY |
| Glyma.13G133000 | up | Zinc finger protein ZAT11 | C2H2 |
| Glyma.13G139000 | down | Zinc finger protein JACKDAW | C2H2 |
| Glyma.13G236500 | up | Ethylene-responsive transcription factor 4 | ERF |
| Glyma.13G248000 | up | B-box domain protein 31 | MIP1B |
| Glyma.13G266600 | up | BES1/BZR1 homolog protein 4 | BES1 |
| Glyma.13G291400 | down | Transcription factor bHLH30 | bHLH |
| Glyma.13G298600 | up | Ethylene-responsive transcription factor ERF053 | ERF |
| Glyma.13G315300 | up | NAC domain-containing protein 90 | NAC |
| Glyma.13G370100 | up | Probable WRKY transcription factor 40 | WRKY |
| Glyma.14G016300 | up | Zinc finger CCCH domain-containing protein 29 | C3H |
| Glyma.14G039200 | up | Transcription factor GAMYB | MYB |
| Glyma.14G086500 | up | Transcription factor MYB44 | MYB |
| Glyma.14G088300 | up | Zinc finger protein ZAT10 | C2H2 |
| Glyma.14G147500 | up | Ethylene-responsive transcription factor ERF012 | ERF |
| Glyma.14G171500 | up | Ethylene-responsive transcription factor RAP2-4 | ERF |
| Glyma.14G190400 | down | Zinc finger protein CONSTANS-LIKE 10 | CO-like |
| Glyma.14G200200 | up | WRKY transcription factor WRKY24 | WRKY |
| Glyma.14G217200 | up | Transcription factor VIP1 | bZIP |
| Glyma.15G003300 | up | Probable WRKY transcription factor 40 | WRKY |
| Glyma.15G064500 | up | Transcription factor bHLH123 | bHLH |
| Glyma.15G110300 | up | WRKY transcription factor 6 | WRKY |
| Glyma.15G168800 | up | Homeobox-leucine zipper protein HAT22 | HD-ZIP |
| Glyma.15G234100 | down | Trihelix transcription factor ASIL2 | Trihelix |
| Glyma.16G016400 | up | NAC domain-containing protein 62 | NAC |
| Glyma.16G020500 | up | Transcription factor bHLH14 | bHLH |
| Glyma.16G023900 | up | Transcription factor bHLH13 | bHLH |
| Glyma.16G026400 | up | Probable WRKY transcription factor 41 | WRKY |
| Glyma.16G042300 | down | GATA transcription factor 9 | GATA |
| Glyma.16G199000 | up | Dehydration-responsive element-binding protein 1D | ERF |
| Glyma.17G007600 | up | Scarecrow-like protein 13 | GRAS |
| Glyma.17G011400 | up | WRKY transcription factor 6 | WRKY |
| Glyma.17G058600 | up | Transcription factor bHLH35 | bHLH |
| Glyma.17G131800 | up | Ethylene-responsive transcription factor ERF025 | ERF |
| Glyma.17G131900 | up | Dehydration-responsive element-binding protein 1C | ERF |
| Glyma.17G185000 | up | NAC domain-containing protein 83 | NAC |
| Glyma.17G224800 | up | Probable WRKY transcription factor 50 | WRKY |
| Glyma.17G237900 | up | Transcription factor MYB44 | MYB |
| Glyma.17G255800 | up | Transcription factor VIP1 | bZIP |
| Glyma.18G056600 | up | WRKY transcription factor WRKY24 | WRKY |
| Glyma.18G081100 | up | Scarecrow-like protein 5 | GRAS |
| Glyma.18G208800 | up | Probable WRKY transcription factor 33 | WRKY |
| Glyma.18G213200 | up | Probable WRKY transcription factor 70 | WRKY |
| Glyma.19G195800 | up | NAC domain-containing protein 90 | NAC |
| Glyma.19G218800 | up | Transcription factor MYB78 | MYB |
| Glyma.19G254800 | up | Probable WRKY transcription factor 41 | WRKY |
| Glyma.20G060400 | down | Zinc finger protein CONSTANS-LIKE 9 | CO-like |
| Glyma.20G130200 | up | Transcription factor bHLH113 | bHLH |
| Glyma.20G155100 | up | Dehydration-responsive element-binding protein 1A | ERF |
| Glyma.20G155200 | up | Ethylene-responsive transcription factor ERF025 | ERF |
| Glyma.20G180100 | up | GATA transcription factor 8 | GATA |

| gene_id | Regulation | Description | Family |
| --- | --- | --- | --- |
| Glyma.01G087500 | up | AP2/ERF and B3 domain-containing transcription repressor TEM1 | RAV |
| Glyma.01G128100 | up | Probable WRKY transcription factor 33 | WRKY |
| Glyma.01G206700 | up | Ethylene-responsive transcription factor 6 | ERF |
| Glyma.01G217400 | down | Heat stress transcription factor B-2b | HSF |
| Glyma.01G224800 | up | Probable WRKY transcription factor 41 | WRKY |
| Glyma.02G000800 | up | Transcription factor bHLH92 | bHLH |
| Glyma.02G132500 | up | Ethylene-responsive transcription factor ERF112 | ERF |
| Glyma.03G112700 | up | Ethylene-responsive transcription factor 13 | ERF |
| Glyma.03G130600 | down | Transcription factor ORG2 | bHLH |
| Glyma.03G256700 | up | Probable WRKY transcription factor 41 | WRKY |
| Glyma.04G036700 | up | Transcription factor MYB44 | MYB |
| Glyma.04G218700 | up | Probable WRKY transcription factor 51 | WRKY |
| Glyma.04G238300 | up | Probable WRKY transcription factor 30 | WRKY |
| Glyma.05G184500 | up | Probable WRKY transcription factor 51 | WRKY |
| Glyma.05G215900 | up | Probable WRKY transcription factor 41 | WRKY |
| Glyma.06G036800 | up | Transcription factor MYB44 | MYB |
| Glyma.06G125600 | up | Probable WRKY transcription factor 30 | WRKY |
| Glyma.06G154400 | up | NAC domain-containing protein 104 | NAC |
| Glyma.06G157400 | up | NAC domain-containing protein 2 | NAC |
| Glyma.06G283300 | up | Transcription factor bHLH30 | bHLH |
| Glyma.06G300100 | up | Transcription factor MYB14 | MYB |
| Glyma.06G300200 | up | Transcription factor MYB14 | MYB |
| Glyma.07G023300 | up | Probable WRKY transcription factor 40 | WRKY |
| Glyma.07G057400 | up | Probable WRKY transcription factor 41 | WRKY |
| Glyma.07G113800 | up | Ethylene-responsive transcription factor 2 | ERF |
| Glyma.07G212400 | up | Ethylene-responsive transcription factor ERF109 | ERF |
| Glyma.08G021900 | up | Probable WRKY transcription factor 41 | WRKY |
| Glyma.08G044400 | up | Protein SAR DEFICIENT 1 | SARD1 |
| Glyma.08G323800 | up | Protein ENHANCED DOWNY MILDEW 2 | EDM2 |
| Glyma.09G052800 | up | Ethylene-responsive transcription factor ERF098 | ERF |
| Glyma.09G147200 | up | Dehydration-responsive element-binding protein 1D | ERF |
| Glyma.09G233800 | up | Dehydration-responsive element-binding protein 3 | ERF |
| Glyma.10G205200 | up | Transcription factor TFIIIB component B | bdp1 |
| Glyma.11G096600 | up | NAC domain-containing protein 35 | NAC |
| Glyma.12G022700 | up | NAC domain-containing protein 35 | NAC |
| Glyma.12G104600 | up | Transcription factor MYB14 | MYB |
| Glyma.12G104800 | up | Transcription factor MYB14 | MYB |
| Glyma.12G105400 | up | Transcription factor MYB14 | MYB |
| Glyma.12G186200 | up | NAC domain-containing protein 90 | NAC |
| Glyma.12G198400 | down | B-box domain protein 31 | MIP1B |
| Glyma.12G206900 | down | NAC domain-containing protein 73 | NAC |
| Glyma.12G231500 | up | BES1/BZR1 homolog protein 4 | BES1 |
| Glyma.13G117600 | up | WRKY transcription factor 42 | WRKY |
| Glyma.13G370100 | up | Probable WRKY transcription factor 40 | WRKY |
| Glyma.14G102900 | up | Probable WRKY transcription factor 40 | WRKY |
| Glyma.14G103100 | up | Probable WRKY transcription factor 40 | WRKY |
| Glyma.14G178000 | up | Transcription factor bHLH96 | bHLH |
| Glyma.15G003300 | up | Probable WRKY transcription factor 40 | WRKY |
| Glyma.16G026400 | up | Probable WRKY transcription factor 41 | WRKY |
| Glyma.16G199000 | up | Dehydration-responsive element-binding protein 1D | ERF |
| Glyma.17G222300 | up | Probable WRKY transcription factor 40 | WRKY |
| Glyma.18G056600 | up | WRKY transcription factor WRKY24 | WRKY |
| Glyma.19G213100 | up | Ethylene-responsive transcription factor ERF071 | ERF |
| Glyma.19G254800 | up | Probable WRKY transcription factor 41 | WRKY |
| Glyma.20G168500 | up | Ethylene-responsive transcription factor RAP2-6 | ERF |

T0ZD_R_P VS T1ZD_R_P：

T0ZD_R_S VS T1ZD_R_S：

| gene_id | Regulation | Description | Family |
| --- | --- | --- | --- |
| Glyma.13G266600 | up | BES1/BZR1 homolog protein 4 | BES1 |
| Glyma.01G197900 | up | Transcription factor bHLH25 | bHLH |
| Glyma.02G000800 | up | Transcription factor bHLH92 | bHLH |
| Glyma.04G098400 | up | Transcription factor bHLH96 | bHLH |
| Glyma.04G200500 | up | Transcription factor ICE1 | bHLH |
| Glyma.05G110600 | up | Transcription factor bHLH25 | bHLH |
| Glyma.06G291900 | up | Transcription factor bHLH36 | bHLH |
| Glyma.09G060200 | up | Transcription factor bHLH35 | bHLH |
| Glyma.11G043700 | up | Transcription factor bHLH25 | bHLH |
| Glyma.11G117100 | up | Transcription factor bHLH49 | bHLH |
| Glyma.12G114300 | up | Transcription factor bHLH36 | bHLH |
| Glyma.13G101100 | up | Transcription factor bHLH35 | bHLH |
| Glyma.13G368700 | up | Transcription factor bHLH25 | bHLH |
| Glyma.15G005000 | up | Transcription factor bHLH25 | bHLH |
| Glyma.15G064500 | up | Transcription factor bHLH123 | bHLH |
| Glyma.15G166800 | up | Transcription factor bHLH35 | bHLH |
| Glyma.16G020500 | up | Transcription factor bHLH14 | bHLH |
| Glyma.16G023900 | up | Transcription factor bHLH13 | bHLH |
| Glyma.16G087300 | up | Transcription factor bHLH62 | bHLH |
| Glyma.17G058600 | up | Transcription factor bHLH35 | bHLH |
| Glyma.20G130200 | up | Transcription factor bHLH113 | bHLH |
| Glyma.01G069300 | down | Basic leucine zipper 43 | bZIP |
| Glyma.14G217200 | up | Transcription factor VIP1 | bZIP |
| Glyma.17G255800 | up | Transcription factor VIP1 | bZIP |
| Glyma.18G277100 | down | Basic leucine zipper 43 | bZIP |
| Glyma.01G209100 | up | Zinc finger protein ZAT9 | C2H2 |
| Glyma.02G058500 | down | Protein indeterminate-domain 2 | C2H2 |
| Glyma.05G137000 | up | Protein indeterminate-domain 14 | C2H2 |
| Glyma.08G092300 | up | Protein indeterminate-domain 14 | C2H2 |
| Glyma.10G045400 | up | Zinc finger protein ZAT11 | C2H2 |
| Glyma.11G033100 | up | Zinc finger protein ZAT9 | C2H2 |
| Glyma.11G142500 | up | Zinc finger protein ZAT8 | C2H2 |
| Glyma.12G070300 | up | Protein indeterminate-domain 9 | C2H2 |
| Glyma.13G133100 | up | Zinc finger protein ZAT11 | C2H2 |
| Glyma.13G333400 | up | Zinc finger protein ZAT11 | C2H2 |
| Glyma.14G088300 | up | Zinc finger protein ZAT10 | C2H2 |
| Glyma.14G110900 | down | Zinc finger protein 3 | C2H2 |
| Glyma.17G236200 | up | Zinc finger protein ZAT10 | C2H2 |
| Glyma.19G174200 | up | Zinc finger protein ZAT11 | C2H2 |
| Glyma.19G180400 | up | Zinc finger protein JACKDAW | C2H2 |
| Glyma.03G138600 | up | Zinc finger CCCH domain-containing protein 30 | C3H |
| Glyma.06G050300 | down | Zinc finger CCCH domain-containing protein 23 | C3H |
| Glyma.08G135200 | up | Calmodulin-binding transcription activator 2 | CAMTA |
| Glyma.07G091400 | down | Zinc finger protein CONSTANS-LIKE 16 | CO-like |
| Glyma.02G108600 | down | Cyclic dof factor 3 | Dof |
| Glyma.07G012100 | up | Dof zinc finger protein DOF1.7 | Dof |
| Glyma.07G229600 | down | Cyclic dof factor 2 | Dof |
| Glyma.13G329000 | up | Dof zinc finger protein DOF5.3 | Dof |
| Glyma.13G352000 | up | Dof zinc finger protein DOF1.7 | Dof |
| Glyma.01G147600 | up | Dehydration-responsive element-binding protein 3 | ERF |
| Glyma.01G216000 | up | Dehydration-responsive element-binding protein 1F | ERF |
| Glyma.01G225000 | up | Ethylene-responsive transcription factor ERF061 | ERF |
| Glyma.01G231000 | up | Ethylene-responsive transcription factor ERF022 | ERF |
| Glyma.01G231200 | up | Ethylene-responsive transcription factor ERF021 | ERF |
| Glyma.02G016100 | up | Ethylene-responsive transcription factor RAP2-3 | ERF |
| Glyma.02G080200 | up | Ethylene-responsive transcription factor ERF110 | ERF |
| Glyma.02G132500 | up | Ethylene-responsive transcription factor ERF112 | ERF |
| Glyma.03G112700 | up | Ethylene-responsive transcription factor 13 | ERF |
| Glyma.03G112800 | up | Ethylene-responsive transcription factor 13 | ERF |
| Glyma.03G191800 | up | Ethylene-responsive transcription factor ERF024 | ERF |
| Glyma.04G057700 | up | Ethylene-responsive transcription factor ERF018 | ERF |
| Glyma.04G084000 | up | Dehydration-responsive element-binding protein 3 | ERF |
| Glyma.04G251400 | up | Ethylene-responsive transcription factor ERF012 | ERF |
| Glyma.05G049800 | up | Ethylene-responsive transcription factor ERF026 | ERF |
| Glyma.05G049900 | up | Dehydration-responsive element-binding protein 1E | ERF |
| Glyma.05G186700 | up | Ethylene-responsive transcription factor ABR1 | ERF |
| Glyma.06G058400 | up | Ethylene-responsive transcription factor ERF018 | ERF |
| Glyma.06G111300 | up | Ethylene-responsive transcription factor ERF012 | ERF |
| Glyma.06G125100 | up | Ethylene-responsive transcription factor CRF4 | ERF |
| Glyma.06G295300 | up | Ethylene-responsive transcription factor ERF053 | ERF |
| Glyma.07G113800 | up | Ethylene-responsive transcription factor 2 | ERF |
| Glyma.07G212400 | up | Ethylene-responsive transcription factor ERF109 | ERF |
| Glyma.08G035000 | up | Dehydration-responsive element-binding protein 3 | ERF |
| Glyma.09G147200 | up | Dehydration-responsive element-binding protein 1D | ERF |
| Glyma.09G233800 | up | Dehydration-responsive element-binding protein 3 | ERF |
| Glyma.10G016500 | up | Ethylene-responsive transcription factor RAP2-3 | ERF |
| Glyma.10G036700 | up | Ethylene-responsive transcription factor 1B | ERF |
| Glyma.10G066900 | up | Ethylene-responsive transcription factor ERF024 | ERF |
| Glyma.10G239400 | up | Dehydration-responsive element-binding protein 1D | ERF |
| Glyma.11G014200 | up | Ethylene-responsive transcription factor ERF021 | ERF |
| Glyma.11G019000 | up | Ethylene-responsive transcription factor CRF2 | ERF |
| Glyma.11G199300 | up | Pathogenesis-related genes transcriptional activator PTI6 | ERF |
| Glyma.12G086200 | up | Dehydration-responsive element-binding protein 1F | ERF |
| Glyma.12G110400 | up | Ethylene-responsive transcription factor ERF053 | ERF |
| Glyma.13G081600 | up | Ethylene-responsive transcription factor ERF014 | ERF |
| Glyma.13G151900 | up | Ethylene-responsive transcription factor ERF024 | ERF |
| Glyma.14G147500 | up | Ethylene-responsive transcription factor ERF012 | ERF |
| Glyma.15G025100 | up | Ethylene-responsive transcription factor ERF024 | ERF |
| Glyma.15G077100 | up | Ethylene-responsive transcription factor 4 | ERF |
| Glyma.16G199000 | up | Dehydration-responsive element-binding protein 1D | ERF |
| Glyma.17G131800 | up | Ethylene-responsive transcription factor ERF025 | ERF |
| Glyma.17G131900 | up | Dehydration-responsive element-binding protein 1C | ERF |
| Glyma.17G145400 | up | Ethylene-responsive transcription factor 1A | ERF |
| Glyma.18G018200 | down | Ethylene-responsive transcription factor ERF060 | ERF |
| Glyma.19G192400 | up | Ethylene-responsive transcription factor ERF024 | ERF |
| Glyma.19G256800 | up | Ethylene-responsive transcription factor ERF023 | ERF |
| Glyma.20G155100 | up | Dehydration-responsive element-binding protein 1A | ERF |
| Glyma.20G155200 | up | Ethylene-responsive transcription factor ERF025 | ERF |
| Glyma.01G086700 | up | Myb family transcription factor EFM | G2-like |
| Glyma.01G183700 | up | Myb family transcription factor EFM | G2-like |
| Glyma.03G118100 | up | GATA transcription factor 2 | GATA |
| Glyma.07G108900 | up | GATA transcription factor 2 | GATA |
| Glyma.10G210500 | up | GATA transcription factor 9 | GATA |
| Glyma.12G037700 | up | GATA transcription factor 7 | GATA |
| Glyma.14G145700 | up | GATA transcription factor 9 | GATA |
| Glyma.16G155300 | up | GATA transcription factor 9 | GATA |
| Glyma.20G180100 | up | GATA transcription factor 8 | GATA |
| Glyma.02G074800 | down | Protein SCARECROW | GRAS |
| Glyma.02G297700 | up | Scarecrow-like transcription factor PAT1 | GRAS |
| Glyma.06G265500 | up | Chitin-inducible gibberellin-responsive protein 1 | GRAS |
| Glyma.07G266500 | up | Scarecrow-like protein 13 | GRAS |
| Glyma.09G011800 | up | Scarecrow-like protein 13 | GRAS |
| Glyma.09G133600 | up | Scarecrow-like protein 8 | GRAS |
| Glyma.11G096000 | up | Scarecrow-like protein 3 | GRAS |
| Glyma.12G137700 | up | Chitin-inducible gibberellin-responsive protein 1 | GRAS |
| Glyma.13G285400 | up | Chitin-inducible gibberellin-responsive protein 1 | GRAS |
| Glyma.17G007600 | up | Scarecrow-like protein 13 | GRAS |
| Glyma.18G081100 | up | Scarecrow-like protein 5 | GRAS |
| Glyma.07G052100 | down | Homeobox-leucine zipper protein ATHB-12 | HD-ZIP |
| Glyma.01G185800 | up | Heat shock factor protein HSF24 | HSF |
| Glyma.10G066100 | up | Heat stress transcription factor A-3 | HSF |
| Glyma.11G025700 | down | Heat stress transcription factor B-2b | HSF |
| Glyma.13G151200 | up | Heat stress transcription factor A-3 | HSF |
| Glyma.04G036700 | up | Transcription factor MYB44 | MYB |
| Glyma.05G013000 | down | Transcription factor SRM1 | MYB |
| Glyma.05G098200 | up | Transcription factor MYB44 | MYB |
| Glyma.06G036800 | up | Transcription factor MYB44 | MYB |
| Glyma.06G178600 | up | Transcription factor MYB86 | MYB |
| Glyma.06G299900 | up | Transcription factor MYB14 | MYB |
| Glyma.06G300100 | up | Transcription factor MYB14 | MYB |
| Glyma.06G300200 | up | Transcription factor MYB14 | MYB |
| Glyma.08G317600 | up | Transcription factor GAMYB | MYB |
| Glyma.10G010400 | up | Transcription factor MYB78 | MYB |
| Glyma.11G176500 | up | Transcription factor DIVARICATA | MYB |
| Glyma.12G104600 | up | Transcription factor MYB14 | MYB |
| Glyma.12G105400 | up | Transcription factor MYB14 | MYB |
| Glyma.14G063400 | up | Transcription factor MYB1 | MYB |
| Glyma.14G086500 | up | Transcription factor MYB44 | MYB |
| Glyma.17G121000 | down | Transcription factor SRM1 | MYB |
| Glyma.17G237900 | up | Transcription factor MYB44 | MYB |
| Glyma.18G065200 | up | Transcription factor DIVARICATA | MYB |
| Glyma.19G218800 | up | Transcription factor MYB78 | MYB |
| Glyma.09G167900 | down | Protein REVEILLE 7 | MYB_related |
| Glyma.10G048500 | down | Protein REVEILLE 8 | MYB_related |
| Glyma.12G104800 | up | Transcription factor MYB14 | MYB_related |
| Glyma.13G333200 | down | Transcription factor MYB48 | MYB_related |
| Glyma.15G041100 | down | Transcription factor MYB48 | MYB_related |
| Glyma.16G217700 | down | Protein REVEILLE 7 | MYB_related |
| Glyma.03G197900 | up | NAC domain-containing protein 90 | NAC |
| Glyma.04G208300 | up | NAC domain-containing protein 2 | NAC |
| Glyma.04G212000 | up | NAC domain-containing protein 104 | NAC |
| Glyma.04G249000 | up | NAC domain-containing protein 2 | NAC |
| Glyma.05G195000 | up | NAC domain-containing protein 2 | NAC |
| Glyma.07G047900 | up | NAC domain-containing protein 62 | NAC |
| Glyma.11G075400 | up | NAC domain-containing protein 37 | NAC |
| Glyma.11G096600 | up | NAC domain-containing protein 35 | NAC |
| Glyma.12G022700 | up | NAC domain-containing protein 35 | NAC |
| Glyma.12G091200 | up | NAC domain-containing protein 90 | NAC |
| Glyma.12G186200 | up | NAC domain-containing protein 90 | NAC |
| Glyma.16G016400 | up | NAC domain-containing protein 62 | NAC |
| Glyma.16G152100 | up | NAC domain-containing protein 79 | NAC |
| Glyma.19G056400 | up | SUPPRESSOR OF GAMMA RESPONSE 1 | NAC |
| Glyma.19G195800 | up | NAC domain-containing protein 90 | NAC |
| Glyma.08G165700 | up | Nuclear transcription factor Y subunit C-2 | NF-YC |
| Glyma.03G143100 | up | Squamosa promoter-binding-like protein 9 | SBP |
| Glyma.11G251500 | down | Squamosa promoter-binding-like protein 2 | SBP |
| Glyma.04G029200 | up | BEL1-like homeodomain protein 11 | TALE |
| Glyma.10G057400 | down | Transcription factor TCP11 | TCP |
| Glyma.12G168300 | up | Transcription factor TCP8 | TCP |
| Glyma.01G121000 | up | Trihelix transcription factor GT-3a | Trihelix |
| Glyma.01G128100 | up | Probable WRKY transcription factor 33 | WRKY |
| Glyma.01G224800 | up | Probable WRKY transcription factor 41 | WRKY |
| Glyma.02G232600 | up | WRKY transcription factor WRKY24 | WRKY |
| Glyma.03G042700 | up | Probable WRKY transcription factor 33 | WRKY |
| Glyma.03G159700 | up | Probable WRKY transcription factor 21 | WRKY |
| Glyma.03G256700 | up | Probable WRKY transcription factor 41 | WRKY |
| Glyma.04G223300 | up | Probable WRKY transcription factor 70 | WRKY |
| Glyma.04G238300 | up | Probable WRKY transcription factor 30 | WRKY |
| Glyma.05G215900 | up | Probable WRKY transcription factor 41 | WRKY |
| Glyma.06G061900 | up | Probable WRKY transcription factor 40 | WRKY |
| Glyma.06G125600 | up | Probable WRKY transcription factor 30 | WRKY |
| Glyma.06G142000 | up | Probable WRKY transcription factor 70 | WRKY |
| Glyma.07G023300 | up | Probable WRKY transcription factor 40 | WRKY |
| Glyma.07G057400 | up | Probable WRKY transcription factor 41 | WRKY |
| Glyma.07G262700 | up | WRKY transcription factor 6 | WRKY |
| Glyma.08G021900 | up | Probable WRKY transcription factor 41 | WRKY |
| Glyma.08G142400 | up | Probable WRKY transcription factor 51 | WRKY |
| Glyma.08G218600 | up | Probable WRKY transcription factor 40 | WRKY |
| Glyma.13G117600 | up | WRKY transcription factor 42 | WRKY |
| Glyma.13G370100 | up | Probable WRKY transcription factor 40 | WRKY |
| Glyma.14G200200 | up | WRKY transcription factor WRKY24 | WRKY |
| Glyma.15G003300 | up | Probable WRKY transcription factor 40 | WRKY |
| Glyma.15G110300 | up | WRKY transcription factor 6 | WRKY |
| Glyma.16G026400 | up | Probable WRKY transcription factor 41 | WRKY |
| Glyma.16G177000 | down | Probable WRKY transcription factor 27 | WRKY |
| Glyma.17G011400 | up | WRKY transcription factor 6 | WRKY |
| Glyma.17G035400 | up | Probable WRKY transcription factor 48 | WRKY |
| Glyma.17G168900 | up | Probable WRKY transcription factor 15 | WRKY |
| Glyma.17G224800 | up | Probable WRKY transcription factor 50 | WRKY |
| Glyma.18G208800 | up | Probable WRKY transcription factor 33 | WRKY |
| Glyma.19G254800 | up | Probable WRKY transcription factor 41 | WRKY |
| Glyma.01G032600 | up | Transcription factor EMB1444 | EMB1444 |
| Glyma.05G237200 | up | Protein SAR DEFICIENT 1 | SARD1 |
| Glyma.07G020400 | down | B-box zinc finger protein 32 | BBX32 |
| Glyma.07G093900 | up | Protein SAR DEFICIENT 1 | SARD1 |
| Glyma.08G044400 | up | Protein SAR DEFICIENT 1 | SARD1 |
| Glyma.09G182400 | up | Protein SAR DEFICIENT 1 | SARD1 |
| Glyma.12G056000 | down | Serine/threonine-protein kinase UCN | UNC |
| Glyma.13G250100 | up | Transcription factor DYT1 | DYT1 |
